# One-pot synthesis of new alkyl 1-naphthoates bearing quinoline, pyranone and cyclohexenone moieties *via* metal-free sequential addition/oxidation reactions†

**DOI:** 10.1039/d1ra07092d

**Published:** 2021-11-16

**Authors:** Seyedeh Hekmat Mousavi, Mohammad Reza Mohammadizadeh, Samira Poorsadeghi, Satoru Arimitsu, Fatemeh Mohammadsaleh, Genta Kojya, Shinichi Gima

**Affiliations:** Department of Chemistry, Faculty of Nano and Bioscience and Technology, Persian Gulf University Bushehr 75169 Islamic Republic of Iran f.mohammadsaleh@gmail.com +98 7731 223348; Department of Chemistry, Biology and Marine Science, Faculty of Science, University of the Ryukyus 1-Senbaru, Nakagami Nishihara Okinawa 903-0213 Japan; Center for Research Advancement and Collaboration, University of the Ryukyus Senbaru 1 Nishihara Okinawa 903-0213 Japan

## Abstract

A mild and one-pot synthetic pathway was successfully developed for the synthesis of new naphthoate-based scaffolds containing quinoline, pyranone and cyclohexenone moieties *via* a multistep reaction between acenaphthoquinone and various 1,3-diketones in the presence of different primary aliphatic and benzylic alcohols. This reaction proceeds *via* a sequential addition/oxidation mechanistic process including a metal-free addition step of acenaphthoquinone and 1,3-diketones followed by the H_5_IO_6_-mediated C–C oxidative cleavage of the corresponding vicinal diols at room temperature. The alcohols play a dual role, as the reaction solvent as well as the nucleophile, to conduct the reaction process toward naphthoate formation. All alkyl naphthoate derivatives prepared in this work are new compounds and were definitively characterized using ^1^H-NMR, ^13^C-NMR and HRMS analysis, while X-ray crystallography was carried out for one of the products. The synthesis of a naphthalene-based nucleus attached to heterocyclic moieties is noteworthy to follow in the near future for diverse applications in biology, medicine, metal complex design, and semiconductor and optical materials.

## Abstract 2

Various new alkyl 1-naphthoates bearing quinoline, pyranone and cyclohexenone moieties were successfully synthesized by a one-pot sequential addition/oxidation process.

## Graphical abstract

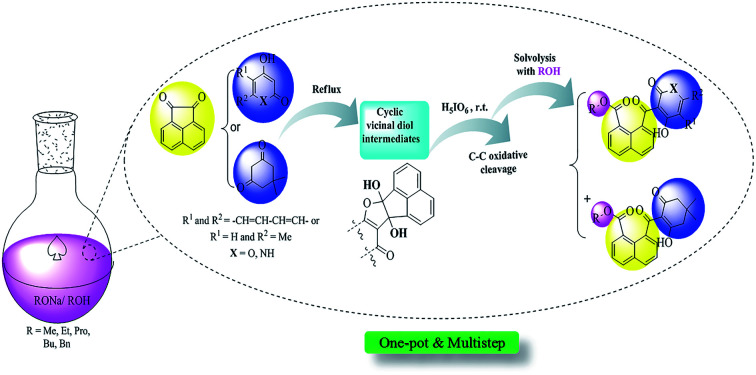

## Introduction

Naphthalene and its derivatives are important organic platforms which are used extensively in industrial chemical compounds and in the chemistry of various pharmacology agents including anticancer,^1,2^ antiviral^3^ and anti-inflammatory^4^ agents and the production of synthetic plastics,^5^ organic semiconductor materials^6^ and optics.^7^

**Fig. 1:**
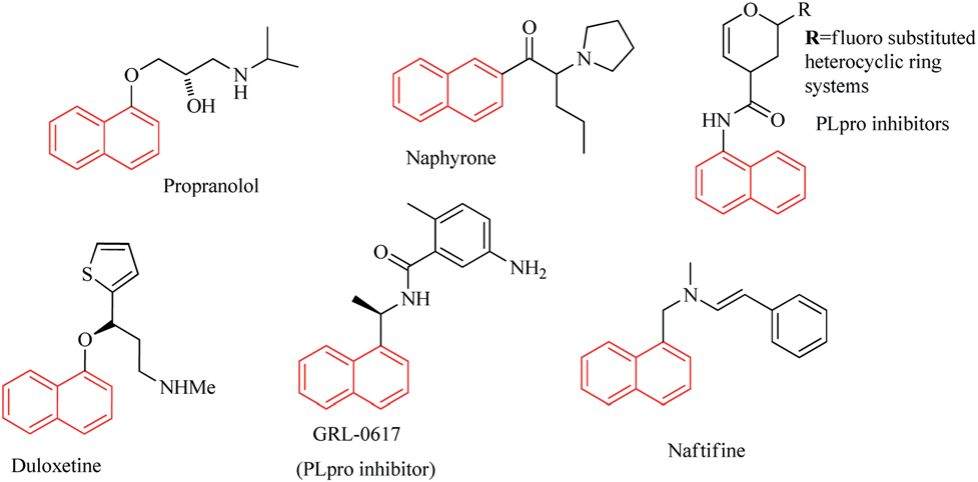
Structural examples for some naphthalene containing drugs.

Heterocyclic compounds are of very much interest to medicinal chemists and many heterocyclic scaffolds have demonstrated unique biological properties.^8–10^ Due to their applications in various fields of chemistry and material sciences,^4^ naphthalene-based heterocyclic compounds have attracted many attentions. Large number of heterocyclic naphthalene-based scaffolds has shown significant and satisfactory biological action.^11–13^ Several naphthalene-based structures have been marketed as medicine and approved by FDA^14,15^ (Fig. 1). However, much studies need to be conducted to the synthesis of new naphthalene scaffolds and the potential of these compounds should be more discovered through extensive research.

Bhati has recently designed a series of naphthalene based SARS-CoV PLpro inhibitors by linking naphthalene scaffold to the 3,4-dihydro-2*H*-pyran moiety *via* –NHCO functional group.^16^ PLpro is an essential enzyme for coronaviruses to express and replicate their genomic and it is very important for targeting PLpro to treat coronavirus infections. The ongoing global pandemic of coronavirus disease 2019 (COVID-19) has influenced all the sections of the society and as the most immediate problem of the world has created an excessive social economic and health care challenge.

Some recent literatures have reported the naphthalene-based PLpro inhibitors and confirmed their potential as COVID-19 therapeutics.^15–20^ Naphthalene based PLpro inhibitors were found to be effective at blocking SARS-CoV-2 (COVID-19) PLpro activity as well as SARS-CoV-2 replication.^21^

Ersan reported the synthesis of new naphthalene utilizing heterocyclic moieties and studied their antimicrobial activity.^11^ These compounds showed high potential in designing new inhibitors of *E. coli* topoisomerase I. Naphthoates as important derivatives of naphthalene exist in many natural and synthetic biologically active materials and their derivatives demonstrate various applications including biological activity,^22^ optoelectronic properties,^23^ ligand and metal-chelator.^3,24^ Liou prepared several methyl naphthalene carboxylates (naphthoates) and evaluated their anti-inflammatory activities by superoxide anion generation and elastase release.^25^ New derivatives of naphthoates have been widely used as ligands and efficient chelators for metal-complex formation with a variety of metals including Cu,^26,27^ Cd,^28,29^ Pb^24^ and *etc.* Dai reported the synthesis and X-ray single-crystal structure analysis of new naphthoate-based cadmium(ii) and lead(ii) complexes.^24,29^ Ahmad Irfan^7^ synthesized Schiff based naphthalene compounds and studied the effect of electron donor/acceptor groups on the electro-optical, charge transfer and NLO properties of products by DFT and TDDFT. Due to the electron injection, electronic coupling constant and light harvesting efficiency they concluded that the studied Schiff base compounds would be good contestants to be used in dye-sensitized solar cells. Based on these results, the heterocyclic scaffolds containing naphthalene ring as fully π-conjugated materials are good candidate for optoelectronic applications and organic semiconductor materials. Chen^26^ prepared the naphthoate-modifying Cu^2+^-detective Bodipy sensors with the fluorescent ON–OFF performance. Their results reveal the effect of the naphthoated esterification on the fluorescence emission of the products.

**Scheme 1:**
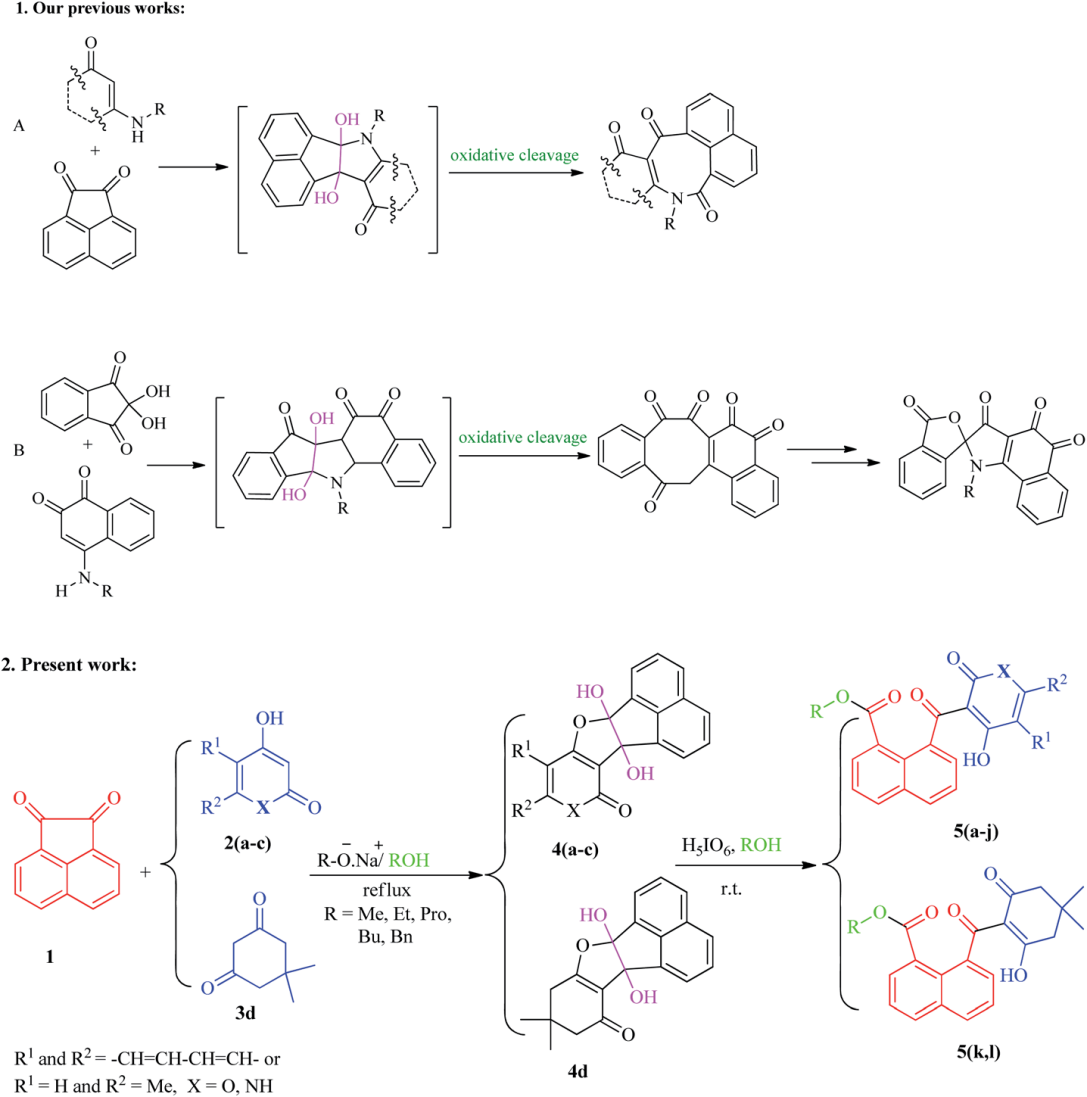
Our synthetic methods on the preparation of various heterocyclic and non-heterocyclic compounds including the C–C oxidative cleavage of cyclic vicinal diols intermediates.

With these considerations in mind, and in the continuation of our efforts to develop new pathways toward the chemical synthesis^30–36^ and as part of our current study on the oxidative cleavages of cyclic vicinal diols to synthesize new potentially biologically active heterocycles,^31^ we have herein focused on the development of a facile and effective method for the synthesis of new naphthoate derivatives (Scheme 1). In this study, we report the results of our efforts on the synthesis of various new alkyl 1-naphthoates containing 2,4-dihydroxyquinoline, 4-hydroxy-2-pyranone, and 2-hydroxycyclohexenones, starting from acenaphthoquinone and various 1,3-diketones in the presence of different primary alcohols (ROH, R: aliphatic and benzylic groups) as solvent and reagent followed by metal-free H_5_IO_6_-mediated oxidative cleavage of the corresponding vicinal diols. The different naphthoate derivatives bearing N- or O-heterocyclic and non-heterocyclic parts were synthesized with good to excellent yields under mild reaction conditions.

## Results and discussion

As shown in the Scheme 1, the various naphthoate derivatives (5a–l) were synthesized in two sequential steps involving addition step resulting to the formation of vicinal cyclic dihydroxy intermediates 4 and C–C oxidative cleavage process as a one-pot reaction. In the addition step, acenaphthoquinone 1 reacted with various derivatives of β-diketones 2 and 3 in different aliphatic and benzylic alcohols under reflux conditions and in the presence of sodium alkoxide (conjugate base of each alcohol).

As described in our previous works, vicinal diols are produced as the main intermediates in the addition reactions between different dinucleophiles and diketones.^31,37^ The C–C bond oxidative cleavage of vicinal diols has been studied in many literatures.^38–40^ Vicinal diols are cleaved by oxo-donor reagents such as periodic acid to yield carbonyl-containing derivatives *via* oxo-transfer mechanism, depending on the reaction conditions, reagents, and the number of groups substituted on the carbon atoms bearing the hydroxyl groups. In this study, no attempts were carried out for separation of diols intermediates 4, thus, after the addition step, they directly treated with H_5_IO_6_ as the oxidant reagent and the reaction was allowed to continue at room temperature.

**Scheme 2:**
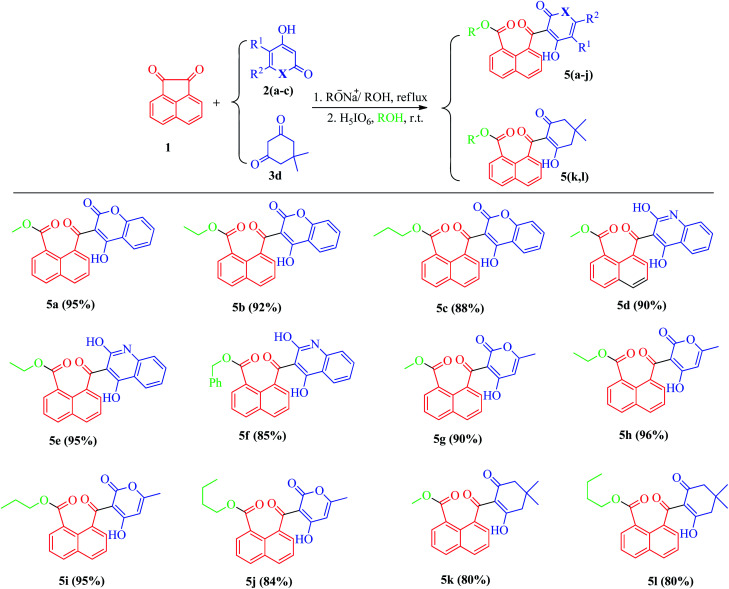
Reaction conditions: β-diketones (1 mmol), acenaphthoquinone (1 mmol); step 1: Na (0.3 mmol), ROH (3 ml), reflux, 12 h; step 2: H_5_IO_6_ (1.1 mmol), r.t., ROH, 30–60 min.

Both reaction steps were performed as one-pot in the presence of various alcohols, in which the alcohol plays a dual role as the reaction solvent as well as nucleophiles after the oxidative cleavage stage to produce the final naphthoate derivatives. Both aliphatic and benzylic alcohols were found to be successful in this reaction process and resulted in the formation of different naphthoate products (5a–l) (Scheme 2).

**Scheme 3:**
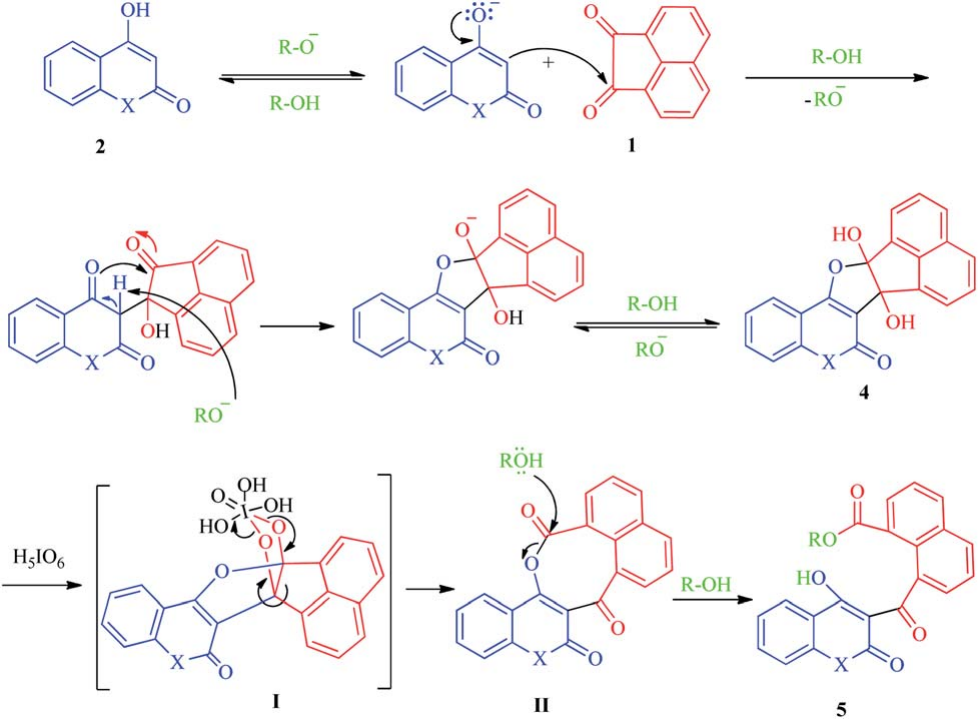
Proposed mechanism for the synthesis of naphthoate derivatives 5.

We also studied the scope and limitation of this synthetic methodology using different cyclic β-dicarbonyls such as dimedone (5k, l), 4-hydroxycoumarin (5a–c), 4-hydroxyquinoline (5d–f) and 4-hydroxy-6-methyl-2-pyrone (5g–j). As shown in Scheme 3, the corresponding naphthoate derivatives were successfully obtained in good to excellent yields, indicating that the type and structure of β-dicarbonyls had no significant effect on the reaction rates, however, under the same reaction conditions, the use of heterocyclic β-dicarbonyls 4-hydroxycoumarin, 4-hydroxyquinoline and 4-hydroxy-6-methyl-2-pyrone gave higher yields of the corresponding products (5a–j) than those for dimedone (5k, l). All the final products were purified by recrystallization and fully characterized by recording their spectral data using ^1^H-NMR, ^13^C-NMR as well as high-resolution mass (HRMS) analysis.

**Fig. 2:**
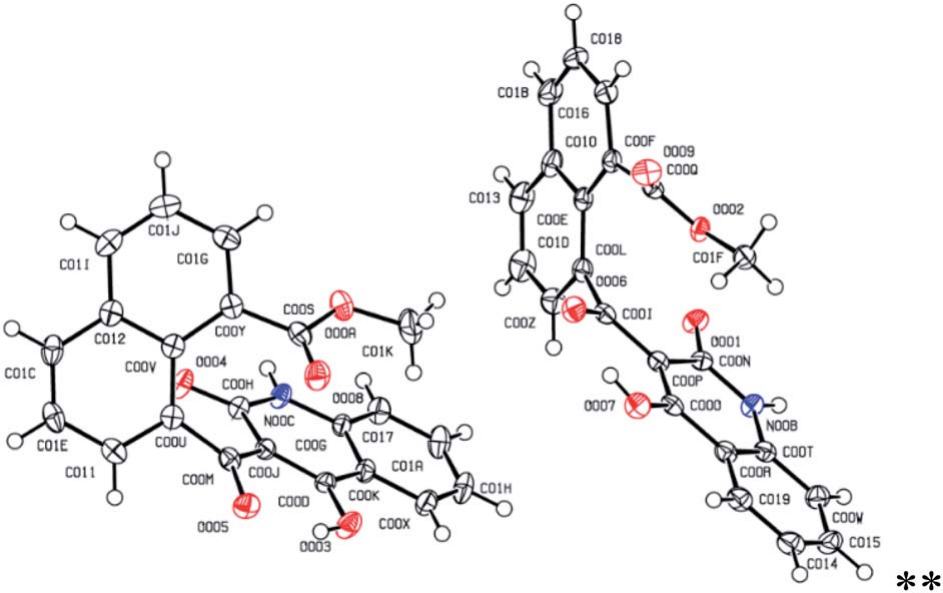
Single-crystal X-ray representation of 5d.

For a typical example, the ^1^H-NMR spectrum of 5d in CDCl_3_ displayed three singlets, at 3.64, 11.52, and 15.92 ppm, which were attributed to the methoxy and the OH groups of quinolone ring, respectively. The aromatic protons were also characterized by the presence of two doublets (*δ* = 6.48, *J* = 8.4 Hz; *δ* = 7.68, *J* = 7.2 Hz), two triplets (*δ* = 7.19, *J* = 7.6 Hz; *δ* = 7.41, *J* = 8.0), one doublet–doublet (*δ* = 7.97, *J* = 7.2 Hz, *J* = 1.6 Hz) and two multiplet peaks at *δ* = 7.52–7.57 and *δ* = 8.03–8.13 ppm. The ^13^C-NMR spectrum of 5d exhibited 22 characteristic signals, whereas methoxy carbon at *δ* = 52.9 and carbonyl carbons were observed at *δ* 169.4 and *δ* 201.8. The aromatic carbons were appeared at the region of 114.6–140.7. The mass analysis of 5d displayed an [M + H]^+^ peak at *m*/*z* = 374.0964, which is in agreement with the proposed structure. We also recorded a single crystal X-ray crystallographic analysis of compound 5d for unambiguous structure determination, which demonstrated the exact structure of this product (Fig. 2).

According to our results and the other reports,^31,41^ we have proposed a plausible mechanism for this synthetic process as depicted in Scheme 3, in which the alkoxide ion acts as the base to activate the 1,3-diketone 2, which subsequently attacks acenaphthoquinone 1 to produce the vicinal diol intermediate 4. Next, the vicinal hydroxyl containing intermediate 4 is oxidatively cleaved by periodic acid to form the intermediate II passed from furo[2,3-*c*][1,2,5]iodadioxole-2,2,2-triol intermediate I.^42^ Due to sp^2^ hybridization of all carbon atoms, specially, existence of rigid acenaphthylene moiety, the intermediate II suffers from significant strain and prone to ring-opening reactions. Thus, it undergoes solvolysis with ROH to produce the final naphthoate derivatives 5. Due to the growing importance of naphthalene-containing compounds in many areas of chemistry and biology, the development of high-yielding and selective synthetic procedures for the synthesis of these versatile platforms will be significantly considered by researchers.

## Conclusions

A variety of new naphthoate derivatives was synthesized *via* a mild, eco-friendly, high-yielding and efficient methodology, in which the various 1,3-diketones reacted with acenaphthoquinone by the sequential addition/oxidation mechanistic reaction process in the presence of RO^−^/ROH. The reaction processes were conducted as metal-free one-pot reaction in two steps. After the addition stage which performed in alcoholic solvents under reflux, the oxidative cleavage step was carried out in the presence of periodic acid at room temperature. The ROH acts as a solvent as well as *O*-nucleophiles and conducts the reaction to the formation of the products. The new naphthoates were purified and fully characterized. In general, reaction rate, reaction condition and selectivity of the products are important advantages that must be considered in this transformation.

## Experimental

### General information

The chemicals used in this work were purchased from Merck and Sigma-Aldrich chemical companies and were used without further purification. Melting points were determined using an Electro thermal 9100 apparatus. ^1^H-NMR and ^13^C-NMR spectra were recorded by using a Bruker DRX-400 AVANCE spectrometer in CDCl_3_ as solvent. IR spectra were recorded using a Shimadzu IR-470 spectrometer with KBr plates.

### General procedure for the synthesis of naphthoate derivatives (5a–l)

To a mixture of sodium (0.3 mmol) and alcohol (3 ml) in a round-bottom flask, 1,3-diketone (1 mmol) and acenaphthoquinone (1 mmol) were added and the mixture was refluxed with stirring for 12 hours. The reaction process was monitored by performing TLC using *n*-hexane/EtOAc. After completion, the reaction mixture was cooled to room temperature and periodic acid (1.1 mmol) was added to the reaction flask and the mixture was stirred at room temperature for about 30–60 min. The final products 5 were formed as the precipitates in the reaction mixture and separated by filtration and recrystallized from their corresponding alcohols to yield pure naphthoate derivatives 5.

## Conflicts of interest

All authors declare that they have no conflict of interest associated with this publication.

## Supplementary Material

RA-011-D1RA07092D-s001

RA-011-D1RA07092D-s002

## References

[cit1] Luo L., Jia J. J., Zhong Q., Zhong X., Zheng S., Wang G., He L. (2021). Eur. J. Med. Chem..

[cit2] Wang G., Qiu J., Xiao X., Cao A., Zhou F. (2018). Bioorg. Chem..

[cit3] Perrone R., Doria F., Butovskaya E., Frasson I., Botti S., Scalabrin M., Lago S., Grande V., Nadai M., Freccero M. (2015). J. Med. Chem..

[cit4] Abozeid M. A., El-Sawi A. A., Abdelmoteleb M., Awad H., Abdel-Aziz M. M., Abdel-Rahman A.-R. H., El-Desoky E.-S. I. (2020). RSC Adv..

[cit5] Hu E., Shang S., Fu Z., Zhao X., Nan X., Du Y., Chen X. (2020). Chemosphere.

[cit6] Kobayashi N., Kuwae H., Oshima J., Ishimatsu R., Tashiro S., Imato T., Adachi C., Shoji S., Mizuno J. (2018). J. Lumin..

[cit7] Irfan A., Kalam A., Chaudhry A. R., Al-Sehemi A. G., Muhammad S. (2017). Optik.

[cit8] Kidwai M., Venktaramanan R., Mohan R., Sapra P. (2002). Curr. Med. Chem..

[cit9] Mermer A., Keles T., Sirin Y. (2021). Bioorg. Chem..

[cit10] Rani S., Raheja K., Luxami V., Paul K. (2021). Bioorg. Chem..

[cit11] Ersan R. H., Yuksel A., Ertan-Bolelli T., Dogen A., Burmaoglu S., Algul O. (2021). J. Chin. Chem. Soc..

[cit12] Park H. J., Song C. W., Sarkar S., Jun Y. W., Reo Y. J., Dai M., Ahn K. H. (2020). Chem. Commun..

[cit13] Chinnasamy G., Subramani K., Srinivasan V. (2017). Orient. J. Chem..

[cit14] Makar S., Saha T., Singh S. K. (2019). Eur. J. Med. Chem..

[cit15] Ghosh A. K., Brindisi M., Shahabi D., Chapman M. E., Mesecar A. D. (2020). ChemMedChem.

[cit16] Bhati S. (2020). Heliyon.

[cit17] Pitsillou E., Liang J., Ververis K., Lim K. W., Hung A., Karagiannis T. C. (2020). Front. Chem..

[cit18] Rao P., Patel R., Shukla A., Parmar P., Rawal R. M., Saraf M., Goswami D. (2021). Mol. Diversity.

[cit19] Shen Z., Ratia K., Cooper L., Kong D., Lee H., Kwon Y., Li Y., Alqarni S., Huang F., Dubrovskyi O. (2021). bioRxiv.

[cit20] Mirza M. U., Ahmad S., Abdullah I., Froeyen M. (2020). Comput. Biol. Chem..

[cit21] Freitas B. T., Durie I. A., Murray J., Longo J. E., Miller H. C., Crich D., Hogan R. J., Tripp R. A., Pegan S. D. (2020). ACS Infect. Dis..

[cit22] Kurosu M., Narayanasamy P., Biswas K., Dhiman R., Crick D. C. (2007). J. Med. Chem..

[cit23] Arunadevi N., Swathika M., Devi B. P., Kanchana P., Sundari S. S., Kirubavathy S. J., Subhapriya P., Kumar E. R. (2021). Surf. Interfaces.

[cit24] Dai P. (2016). Russ. J. Coord. Chem..

[cit25] Liou J.-R., El-Shazly M., Du Y.-C., Tseng C.-N., Hwang T.-L., Chuang Y.-L., Hsu Y.-M., Hsieh P.-W., Wu C.-C., Chen S.-L. (2013). Phytochemistry.

[cit26] Chen Y., Zhao L., Jiang J. (2017). Spectrochim. Acta, Part A.

[cit27] Takasaki Y., Takamizawa S. (2015). Chem. Commun..

[cit28] Arunadevi N., Kanchana P., Hemapriya V., Sankaran S. S., Mayilsamy M., Balakrishnan P. D., Chung I.-M., Mayakrishnan P. (2021). J. Dispersion Sci. Technol..

[cit29] Dai P., Yang E., Zhao X. (2015). Russ. J. Coord. Chem..

[cit30] Mousavi S. H., Mohammadizadeh M. R., Roshan Z., Jamaleddini A., Arimitsu S. (2020). ACS Omega.

[cit31] Mousavi S. H., Mohammadizadeh M. R., Arimitsu S., Saberi D., Poorsadeghi S., Genta K. (2020). RSC Adv..

[cit32] Hashemi S. A., Mohammadizadeh M. R. (2019). ChemistrySelect.

[cit33] Jamaleddini A., Mohammadizadeh M. R. (2017). Tetrahedron Lett..

[cit34] Mohammadizadeh M. R., Saberi D., Taghavi S. Z. (2016). Tetrahedron Lett..

[cit35] Hashemi S. A., Mohammadizadeh M. R. (2021). ChemistrySelect.

[cit36] Firoozi N., Roshan Z., Mohammadizadeh M. R. (2018). Appl. Organomet. Chem..

[cit37] Jamaledini A., Mohammadizadeh M. R. (2018). Heteroat. Chem..

[cit38] Niu T., Chen S., Hong M., Zhang T., Chen J., Dong X., Ni B. (2020). Green Chem..

[cit39] Amadio E., González-Fabra J., Carraro D., Denis W., Gjoka B., Zonta C., Bartik K., Cavani F., Solmi S., Bo C. (2018). Adv. Synth. Catal..

[cit40] Meng L., Li W., Guo P., Wang S., Tong X. (2021). Catal. Commun..

[cit41] Luo H., Wang L., Shang S., Niu J., Gao S. (2019). Commun. Chem..

[cit42] Jackson E. L. (2004). Org. React..

