# Supplementary material for: One-pot synthesis of new alkyl 1-naphthoates bearing quinoline, pyranone and cyclohexenone moieties via metal-free sequential addition/oxidation reactions
Source: RSC Adv. 2021 Nov 16;11(58):36748–52. doi: 10.1039/d1ra07092d (PMC9043593; doi:10.1039/d1ra07092d)
Supplement: RA-011-D1RA07092D-s001 [file RA-011-D1RA07092D-s001.pdf]

Electronic Supplementary Material (ESI) for RSC Advances.
This journal is © The Royal Society of Chemistry 2021

Supporting information

One-pot Synthesis of New Alkyl 1-Naphthoates Bearing Quinoline, Pyranone and Cyclohexenone Moieties *via* the Metal-free Sequential Addition/Oxidation Reactions

Seyedeh Hekmat Mousavi, Mohammad Reza Mohammadizadeh,[a] Samira Poorsadeghi,[b] Satoru Arimitsu,[b] Fatemeh Mohammadsaleh,[a*] Genta Kojya,[c] Shinichi Gima[c]

[a]Department of Chemistry, Faculty of Nano and Bioscience and Technology, Persian Gulf University, Bushehr 75169, (Islamic Republic of Iran), Fax: +98 7731 223348, E-mail: *f.mohammadsaleh@gmail.com*.

[b]Department of Chemistry, Biology and Marine Science, Faculty of Science, university of the Ryukyus, 1-Senbaru, Nishihara, Nakagami, Okinawa, Japan, Zip code: 903-0213.

[c]Center for Research Advancement and Collaboration, University of the Ryukyus, Senbaru 1, Nishihara, Okinawa 903-0213, Japan.

1. The spectral data for the prepared organic compounds

Methyl 8-(4-hydroxy-2-oxo-2*H*-chromene-3-carbonyl)-1-naphthoate (5a)

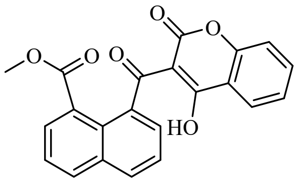

White powder, Mp = 180 °C; ^{1}H-NMR (300 MHz, $CDCl_3$) δ (ppm): 3.74 (s, 3H, OCH_3), 7.41-7.34 (m, 2H, Ar), 7.58-7.54 (m, 2H, Ar), 7.75-7.69 (m, 2H, Ar), 8.08-8.01 (m, 3H, Ar), 8.14 (dd, J_1 = 8 Hz, J_2 = 1.6 Hz, 1H, Ar), 16.67 (s, 1H, OH); ^{13}C-NMR (100 MHz, $CDCl_3$) δ (ppm): 52.1, 101.1, 115.2, 117.2, 124.4, 125.0, 125.2, 125.4, 126.8, 128.3, 128.5, 130.8, 132.0, 133.3, 134.5, 135.6, 136.0, 155.4, 159.0, 169.4, 178.1, 201.7. HRMS (ESI) calculated for $C_{22}H_{14}O_6$ $[M+H]^+$: 375.0869; found: 375.0875.

Ethyl 8-(4-hydroxy-2-oxo-2*H*-chromene-3-carbonyl)-1-naphthoate (5b)

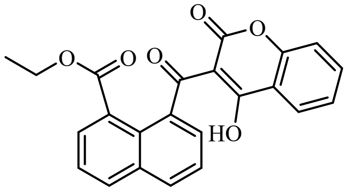

White powder, Mp = 184 °C; ^{1}H-NMR (300 MHz, $CDCl_3$) δ (ppm): 1.28 (t, J = 7.2 Hz, 3H, CH_3), 4.21 (q, J = 7.2 Hz, 3H, OCH_2), 7.33-7.40 (m, 2H, Ar), 7.58-7.53 (m, 2H, Ar), 7.68-7.74 (m, 2H, Ar), 8.00-8.07 (m, 3H, Ar), 8.13 (d, J = 8.0 Hz, 1H, Ar), 16.73 (s, 1H, OH); ^{13}C-NMR (100 MHz, $CDCl_3$) δ (ppm): 14.2, 61.4, 101.3, 115.3, 117.2, 124.3, 125.0, 125.2, 125.4, 126.8,

128.5, 128.6, 130.6, 132.0, 133.2, 134.5, 135.6, 136.0, 155.4, 159.0, 169.1, 178.0, 201.7. HRMS (ESI) calculated for $C_{23}H_{19}O_6$ $[M+H]^+$: 389.1025; found: 389.1025.

Propyl 8-(4-hydroxy-2-oxo-2*H*-chromene-3-carbonyl)-1-naphthoate (5c)

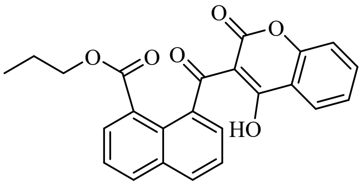

White powder, Mp = 190 °C; ^{1}H-NMR (300 MHz, $CDCl_3$) δ (ppm): 0.94 (t, J = 7.2 Hz, 3H, CH_3), 1.68 (sext, J = 7.2 Hz, 2H, CH_2), 4.12 (t, J = 6.8 Hz, 2H, OCH_2), 7.32-7.40 (m, 2H, Ar), 7.53-7.58 (m, 2H, Ar), 7.67-7.74 (m, 2H, Ar), 7.82 (t, J = 7.6 Hz, 1H, Ar), 8.00-8.07 (m, 2H, Ar), 8.13 (dd, J_1 = 7.6 Hz, J_2 = 1.2 Hz, 1H, Ar), 17.09 (s, 1H, OH); ^{13}C-NMR (100 MHz, $CDCl_3$) δ (ppm): 10.4, 21.9, 67.1, 101.3, 115.3, 117.2, 124.3, 125.0, 125.2, 125.4, 126.8, 128.4, 128.5, 130.5, 131.9, 133.2, 134.5, 135.4, 136.0, 155.3, 159.0, 169.1, 178.1, 201.7.

Methyl 8-(2,4-dihydroxyquinoline-3-carbonyl)-1-naphthoate (5d)

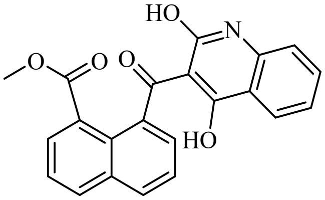

White powder, Mp>230 °C; ^{1}H-NMR (400 MHz, $CDCl_3$) δ (ppm): 3.64 (s, 3H, OCH_3), 6.48 (d, J = 8.4 Hz, 1H, Ar), 7.19 (t, J = 7.6 Hz, 1H, Ar), 7.41 (t, J = 8.0 Hz, 1H, Ar), 7.52-7.57 (m, 2H, Ar), 7.68 (d, J = 7.2 Hz, 1H, Ar), 7.97 (dd, J_1 = 7.2 Hz, J_2 = 1.6 Hz, 1H, Ar), 8.03-8.13 (m, 3H, Ar), 11.52 (s, 1H, OH), 15.92 (s, 1H, OH); ^{13}C-NMR (100 MHz, $CDCl_3$) δ (ppm): 51.9, 105.8, 114.6, 116.1, 122.3, 125.0, 125.1, 125.2, 126.9, 128.1, 128.9, 130.4, 130.9, 132.9, 134.3, 134.4,

137.7, 140.7, 162.6, 169.4, 175.5, 201.8. HRMS (ESI) calculated for $C_{22}H_{15}NO_5$ $[M+H]^+$: 374.1028; found: 374.0964.

Ethyl 8-(2,4-dihydroxyquinoline-3-carbonyl)-1-naphthoate (5e)

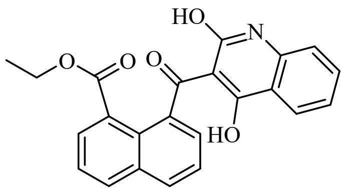

White powder, Mp.>230 °C; ^{1}H-NMR (300 MHz, DMSO-d_6) δ (ppm): 1.07 (t, J = 6.9 Hz, 3H, CH_3), 3.90-4.10 (broad peak, 2H, CH_2), 7.34-7.26 (m, 2H, Ar), 7.62-7.68 (m, 2H, Ar), 7.70-7.75 (m, 2H, Ar), 7.90 (d, J_1 = 6.3 Hz, 1H, Ar), 8.08 (d, J_1 = 8.1 Hz, 1H, Ar), 8.17 (dd, J_1 = 8.2 Hz, J_2 = 1.5 Hz, 1H, Ar), 8.25 (dd, J_1 = 8.0 Hz, J_2 = 1.5 Hz, 1H, Ar), 11.42 (s, 1H, OH), 15.87 (s, 1H, OH); ^{13}C-NMR (100 MHz, DMSO-d_6) δ (ppm): 14.0, 61.3, 106.7,114.0, 116.0, 122.5, 125.2, 125.5, 125.7, 126.6, 128.0, 128.9, 130.1, 131.6, 133.2, 134.2, 135.4, 135.9, 141.8, 160.4, 169.0, 174.7, 201.5.

Benzyl 8-(2,4-dihydroxyquinoline-3-carbonyl)-1-naphthoate (5f)

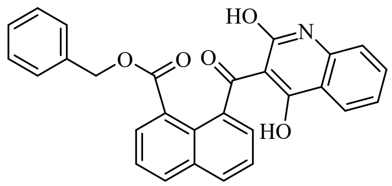

White powder, Mp.>230 °C; ^{1}H-NMR (400 MHz, $CDCl_3$) δ (ppm): 5.11 (s, 2H, OCH_2), 6.52 (d, J = 6.8 Hz, 1H, Ar), 7.11-7.21 (m, 5H, Ar), 7.44 (t, J = 8.0 Hz, 1H, Ar), 7.53-7.56 (m, 2H, Ar), 7.65 (d, J = 4.2 Hz, 1H, Ar), 7.83 (t, J = 8.0 Hz, 1H, Ar), 8.01-8.04 (m, 2H, Ar), 8.08-8.13 (m, 2H, Ar), 10.51 (s, 1H, OH), 15.89 (s, 1H, OH); ^{13}C-NMR (100 MHz, $CDCl_3$) δ (ppm): 60.1,

106.0, 114.6, 115.8, 118.9, 122.2, 125.0, 125.1, 125.2, 126.9, 127.5, 128.0, 128.3, 128.8, 130.5, 130.0, 133.1, 133.5, 134.3, 134.4, 135.3, 140.6, 160.6, 169.0, 175.4, 201.8. HRMS (ESI) calculated for $C_{28}H_{19}NO_5$ $[M+H]^+$: 450.1341; found: 450.1386.

Methyl 8-(4-hydroxy-6-methyl-2-oxo-2H-pyran-3-carbonyl)-1-naphthoate (5g)

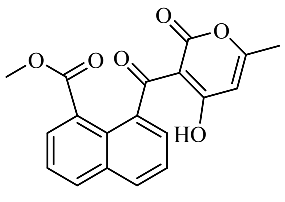

White powder, Mp = 85 °C; ^{1}H-NMR (400 MHz, $CDCl_3$) δ (ppm): 2.31 (s, 3H, CH_3), 3.77 (s, 3H, OCH_3), 6.05 (s, 1H, Ar), 7.51-7.56 (m, 2H, Ar), 7.66 (dd, J_1 = 7.2 Hz, J_2 = 1.2 Hz, 1H, Ar), 7.98-8.05 (m, 3H, Ar), 15.84 (s, 1H, OH); ^{13}C-NMR (100 MHz, $CDCl_3$) δ (ppm): 20.9, 52.0, 99.2, 101.3, 125.0, 125.1, 126.8, 128.4, 128.5, 130.6, 131.8, 133.1, 134.5, 135.6, 160.2, 169.4, 170.0, 181.3, 201.2. HRMS (ESI) calculated for $C_{19}H_{15}O_6$ $[M+H]^+$: 339.0869; found: 339.0866.

Ethyl 8-(4-hydroxy-6-methyl-2-oxo-2*H*-pyran-3-carbonyl)-1-naphthoate (5h)

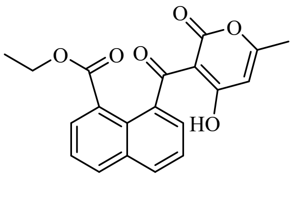

White powder, Mp = 115 °C; ^{1}H-NMR (400 MHz, $CDCl_3$) δ (ppm): 1.33 (t, J = 7.2 Hz, 3H, CH_3-CH_2), 2.31 (s, 3H, CH_3), 4.26 (q, J = 7.2 Hz, 2H, OCH_2), 6.04 (s, 1H, Ar), 7.56-7.51 (m, 2H, Ar), 7.65 (d, J = 8.0 Hz, 1H, Ar), 8.04-7.98 (m, 3H, Ar), 15.90 (s, 1H, OH); ^{13}C-NMR (100 MHz, $CDCl_3$) δ (ppm): 14.2, 20.9, 61.4, 99.4, 101.3, 125.0, 125.1, 126.8, 128.4, 128.8, 130.4, 131.8, 133.0, 134.5, 135.6, 160.2, 169.1, 169.8, 181.3, 201.2. HRMS (ESI) calculated for $C_{20}H_{17}O_6$ $[M+H]^+$: 353.1018; found: 353.1028.

Propyl-8-(4-hydroxy-6-methyl-2-oxo-2*H*-pyran-3-carbonyl)-1-naphthoate (5i)

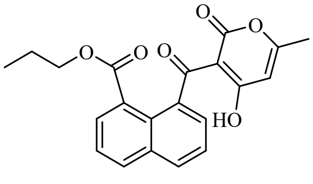

White powder, Mp = 156 °C; ^{1}H-NMR (400 MHz, $CDCl_3$) δ (ppm): 1.01 (t, J= 7.2 Hz, 3H, CH_3-CH_2), 1.74 (sext, J= 7.2 Hz, 2H, CH_2), 2.30 (s, 1H, CH_3), 4.16 (t, , J= 6.8 Hz, 2H, OCH_2), 6.04 (s, 1H, Ar), 7.56-7.51 (m, 2H, Ar), 7.64 (d, J= 7.2 Hz, 1H, Ar), 8.04-7.98 (m, 3H, Ar), 15.92 (s, 1H, OH); ^{13}C-NMR (100 MHz, $CDCl_3$) δ (ppm): 10.5, 20.9, 22.0, 67.0, 99.5, 101.3, 125.0, 125.1, 126.8, 128.4, 128.8, 130.3, 131.8, 133.0, 134.5, 135.6, 160.2, 169.1, 169.8, 181.3, 201.3. HRMS (ESI) calculated for $C_{21}H_{19}O_6$ $[M+H]^+$: 367.1182; found: 367.1185.

Butyl 8-(4-hydroxy-6-methyl-2-oxo-2*H*-pyran-3-carbonyl)-1-naphthoate (5j)

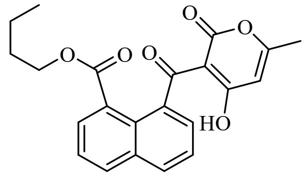

White powder, Mp = 166 °C; ^{1}H-NMR (400 MHz, $CDCl_3$) δ (ppm): 0.95 (t, J= 7.6 Hz, 3H, CH_3-CH_2), 1.44 (sext, J= 8.0 Hz, 2H, CH_2-CH_3), 1.69 (pen, J= 7.2 Hz, 2H, CH_2-CH_2), 2.29 (s, 1H, CH_3), 4.20 (t, , J= 6.8 Hz, 2H, OCH_2), 6.04 (s, 1H, Ar), 7.50-7.55 (m, 2H, Ar), 7.64 (d, J= 7.2 Hz, 1H, Ar), 7.97-8.03 (m, 3H, Ar), 15.91 (s, 1H, OH); ^{13}C-NMR (100 MHz, $CDCl_3$) δ (ppm): 13.7, 19.2, 20.8, 30.6, 65.3, 99.5, 101.3, 125.0, 125.1, 126.8, 128.4, 128.8, 130.3, 131.7, 133.0, 134.5, 135.6, 160.2, 169.1, 169.8, 181.3, 201.2. HRMS (ESI) calculated for $C_{21}H_{19}O_6$ $[M+H]^+$: 381.1321; found: 381.1338.

Methyl 8-(2-hydroxy-4,4-dimethyl-6-oxocyclohex-1-ene-1-carbonyl)-1-naphthoate (5k)

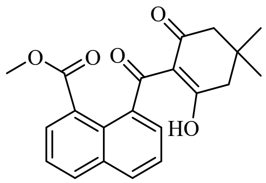

White powder, Mp.>230 °C; ^{1}H-NMR (400 MHz, $CDCl_3$) δ (ppm): 1.16 (s, 6H, CH_3), 2.38 (s, 2H, CH_2), 2.68 (s, 2H, CH_2), 3.75 (s, 3H, OCH_3), 7.49-7.53 (m, 3H, Ar), 7.93-7.97 (m, 2H, Ar), 8.02 (dd, J_1 = 8.0 Hz, J_2 = 1.2 Hz, 1H, Ar), 17.15 (s, 1H, OH); ^{13}C-NMR (100 MHz, $CDCl_3$) δ (ppm): 28.3, 31.2, 46.2, 51.9, 52.0, 112.4, 124.9, 125.0, 126.8, 128.5, 128.7, 130.4, 131.5, 133.0, 134.3, 135.9, 169.5, 193.3, 195.7, 199.3. HRMS (ESI) calculated for $C_{21}H_{21}O_5$ $[M+H]^+$: 353.1389; found: 353.1337.

Butyl 8-(2-hydroxy-4,4-dimethyl-6-oxocyclohex-1-ene-1-carbonyl)-1-naphthoate (5l)

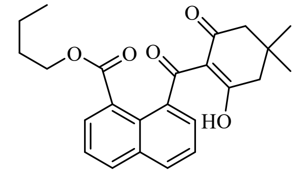

White powder, Mp.>230 °C; ^{1}H-NMR (400 MHz, $CDCl_3$) δ (ppm): 0.95 (t, J= 7.2 Hz, 3H, CH_3-CH_2), 1.15 (s, 6H, CH_3), 1.43 (sext, J= 7.2 Hz, 2H, CH_2-CH_3), 1.70 (pent, J= 6.8 Hz, 2H, CH_2-CH_2), 2.37 (s, 2H, CH_2), 2.66 (s, 2H, CH_2), 4.18 (t, , J= 6.8 Hz, 2H, OCH_2), 7.50-7.46 (m, 3H, Ar), 7.91-7.94 (m, 2H, Ar), 8.00 (dd, J_1 = 8.4 Hz, J_2 = 1.2 Hz, 1H, Ar), 17.22 (s, 1H, OH); ^{13}C-NMR (100 MHz, $CDCl_3$) δ (ppm): 13.7, 19.2, 28.3, 30.6, 31.2, 46.2, 52.0, 65.1, 112.5, 124.9, 125.0, 126.8, 128.4, 129.0, 130.1, 131.3, 132.9, 134.4, 136.0, 169.1, 193.3, 195.7, 199.4. HRMS (ESI) calculated for $C_{24}H_{27}O_5$ $[M+H]^+$: 395.1858; found: 395.1783.

2. The HRMS, ^{1}HNMR and ^{13}CNMR spectra of the prepared organic compounds

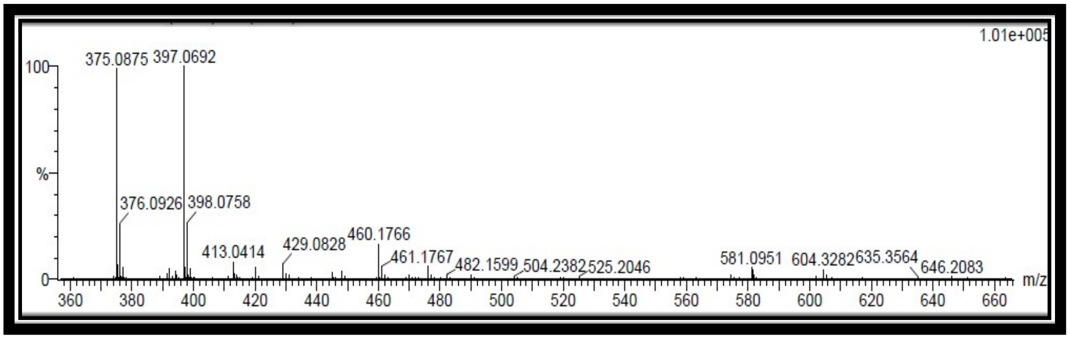

The HRMS spectrum of methyl 8-(4-hydroxy-2-oxo-2*H*-chromene-3-carbonyl)-1-naphthoate **(5a)**

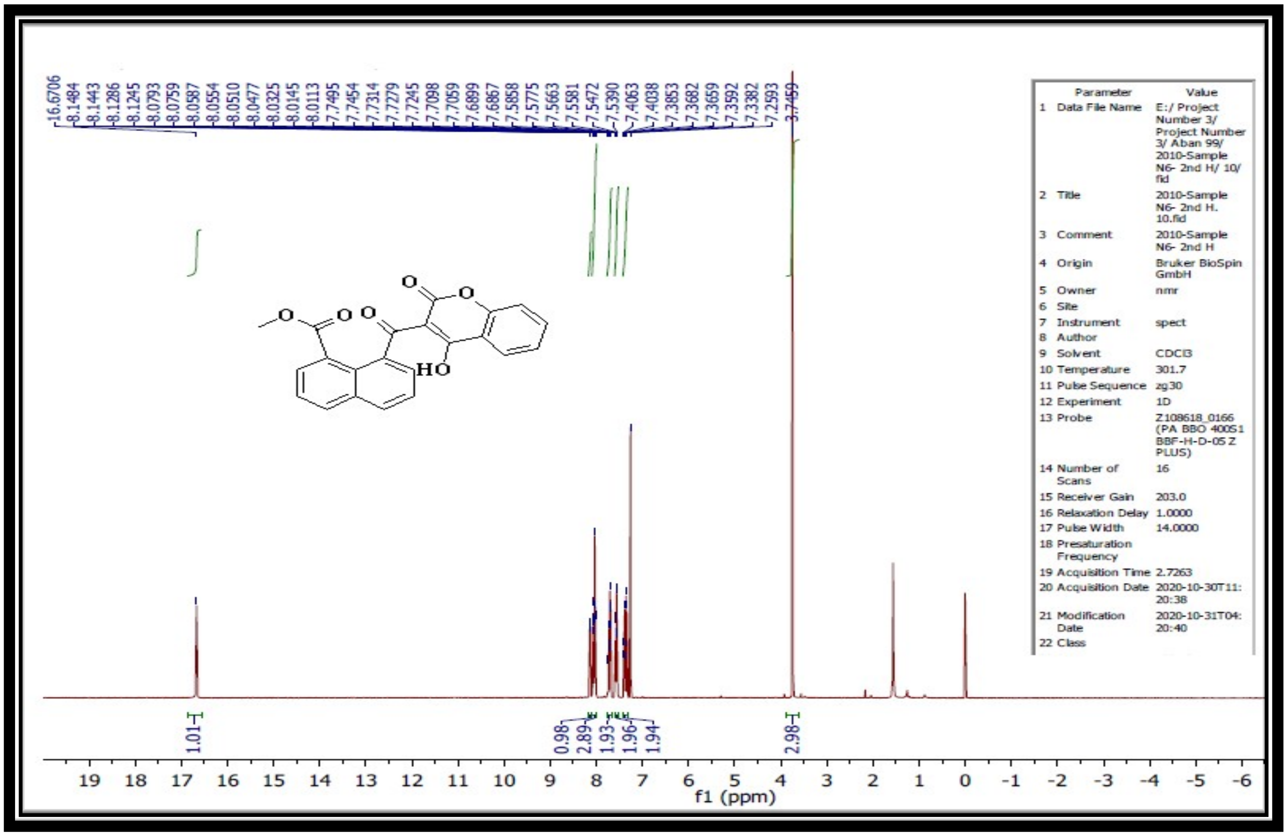

The HNMR spectrum of methyl 8-(4-hydroxy-2-oxo-2*H*-chromene-3-carbonyl)-1-naphthoate **(5a)**

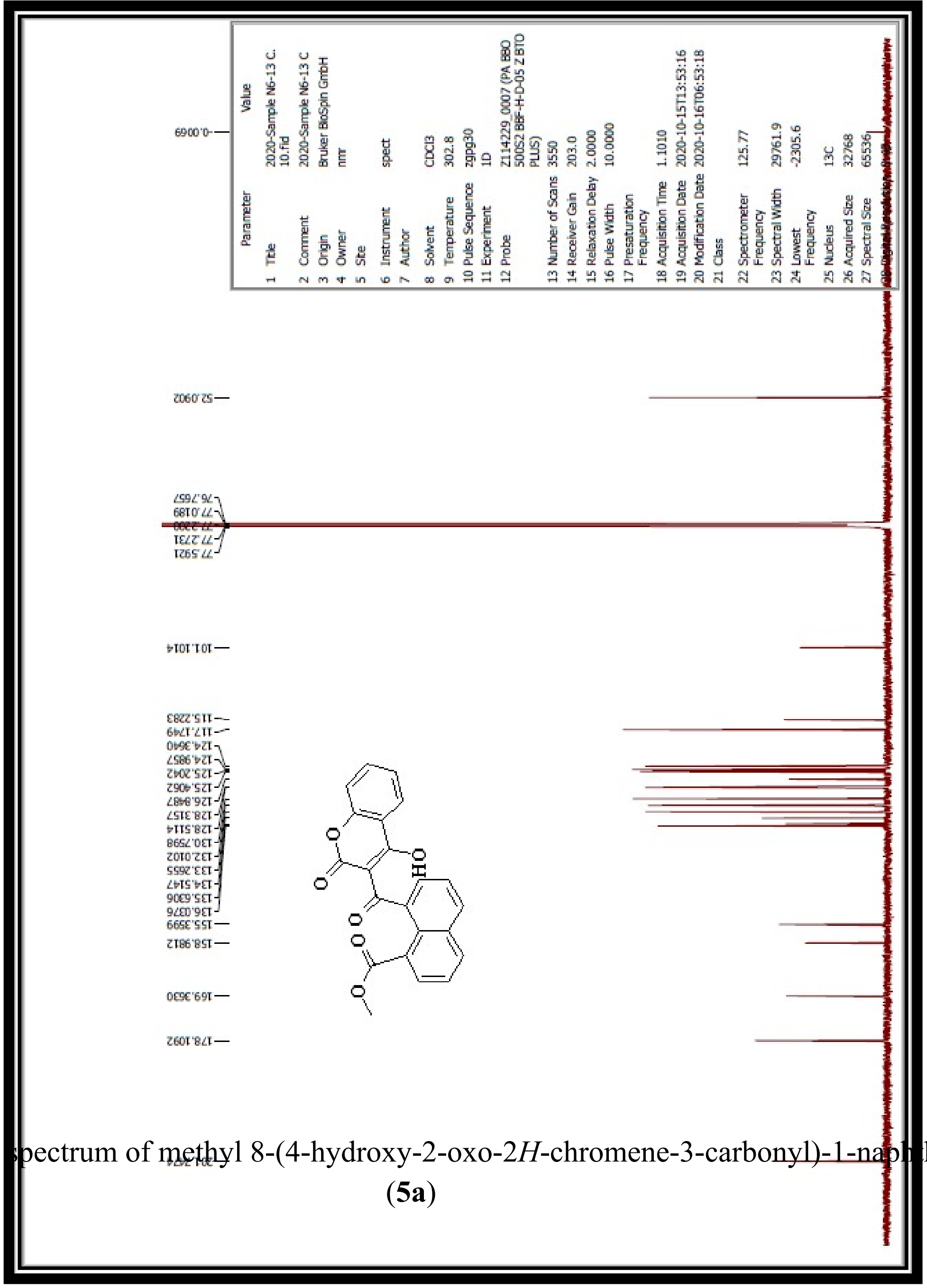

The ^{13}C-NMR spectrum of methyl 8-(4-hydroxy-2-oxo-2*H*-chromene-3-carbonyl)-1-naphthoate (**5a**)

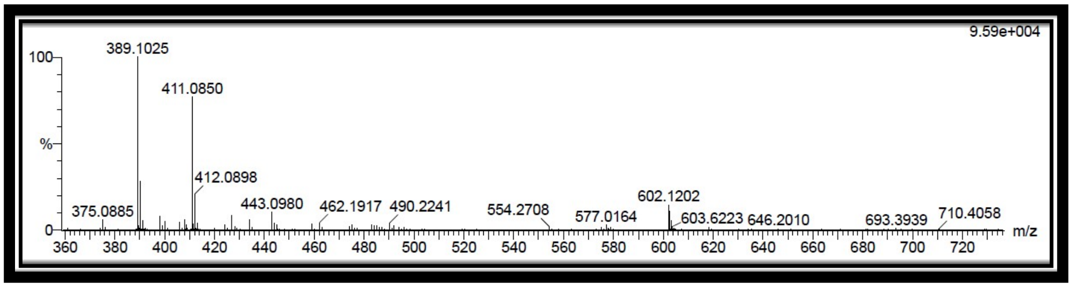

The HRMS spectrum of ethyl 8-(4-hydroxy-2-oxo-2*H*-chromene-3-carbonyl)-1-naphthoate (**5b**)

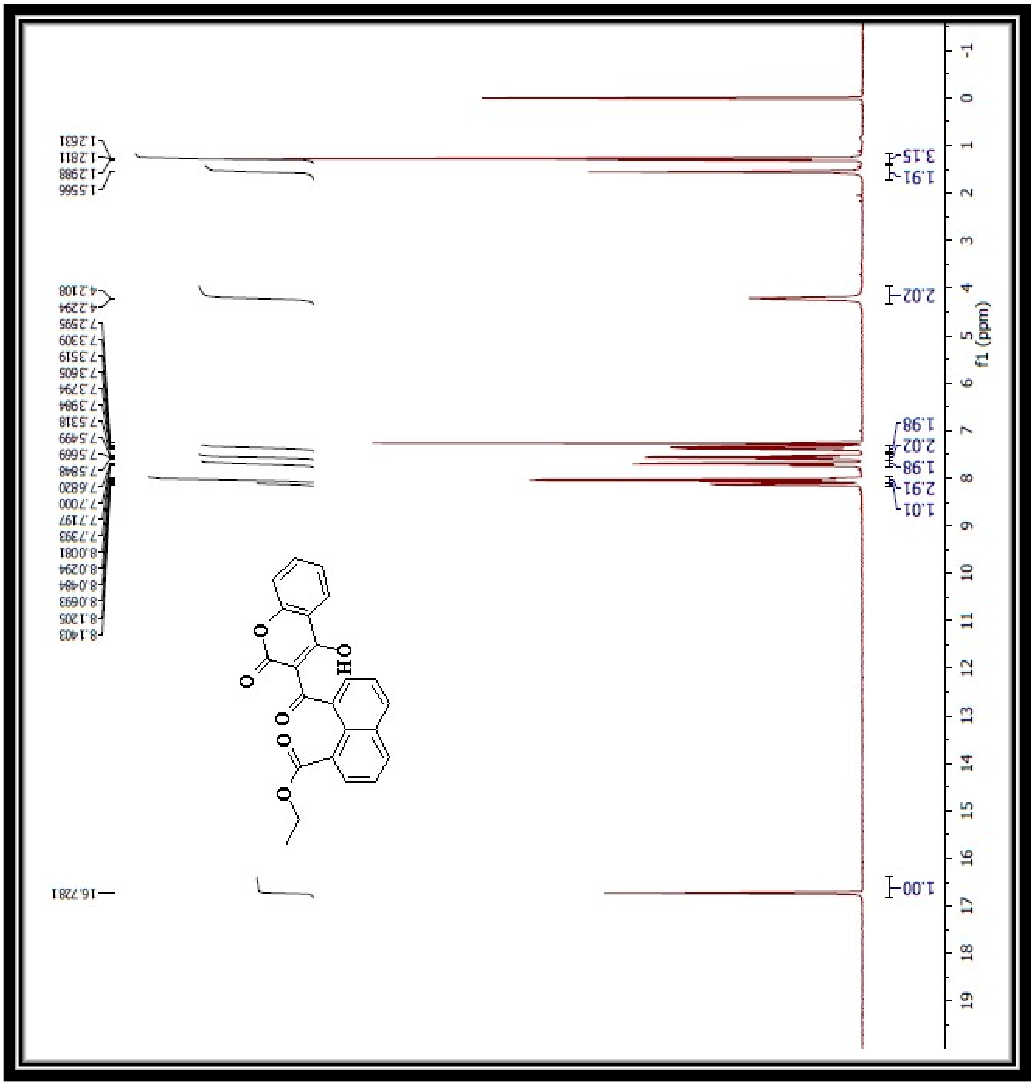

The ^{1}HNMR spectrum of ethyl 8-(4-hydroxy-2-oxo-2*H*-chromene-3-carbonyl)-1-naphthoate (**5b**)

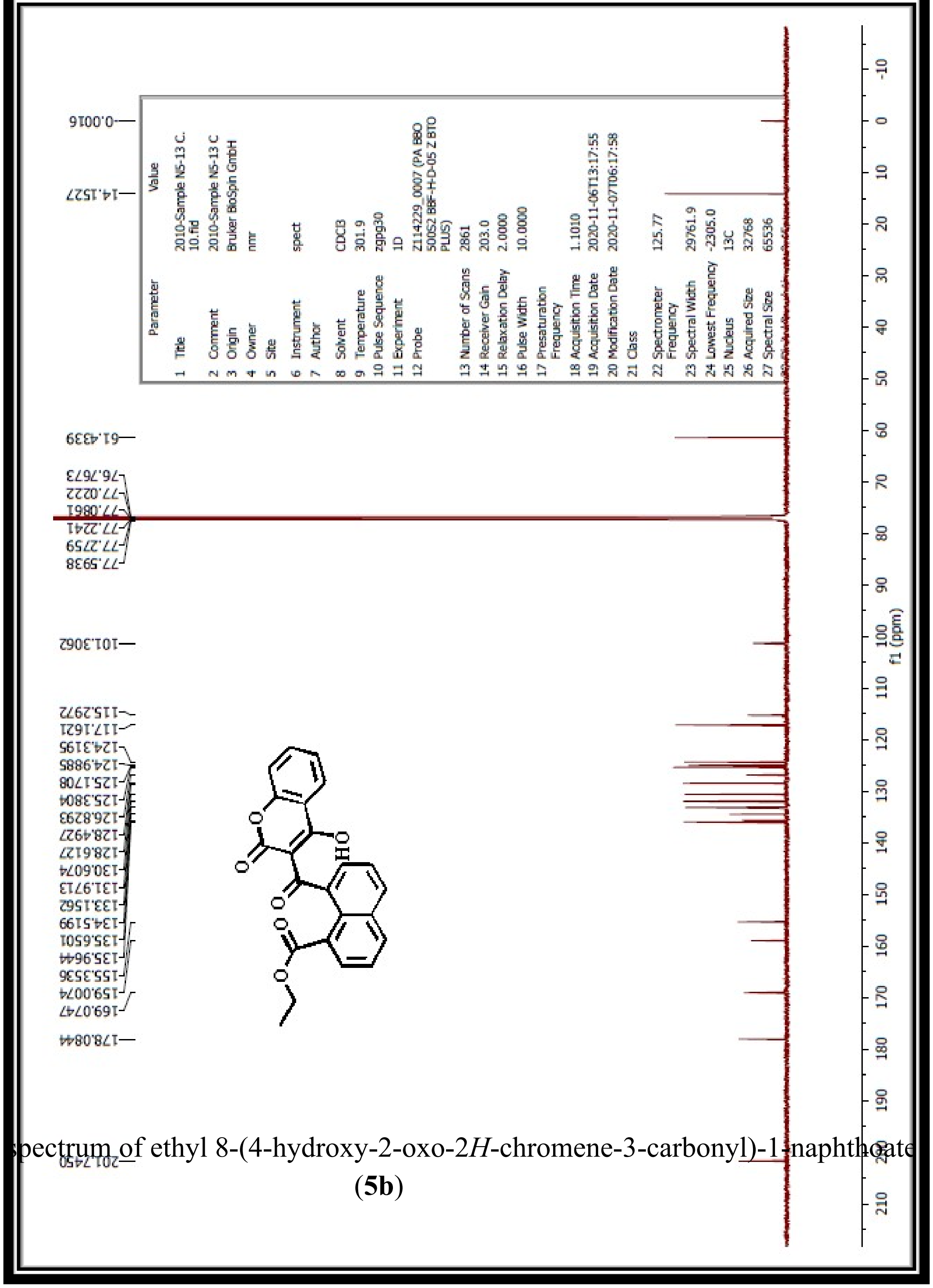

The ^{13}C-NMR spectrum of ethyl 8-(4-hydroxy-2-oxo-2*H*-chromene-3-carbonyl)-1-naphthoate (**5b**)

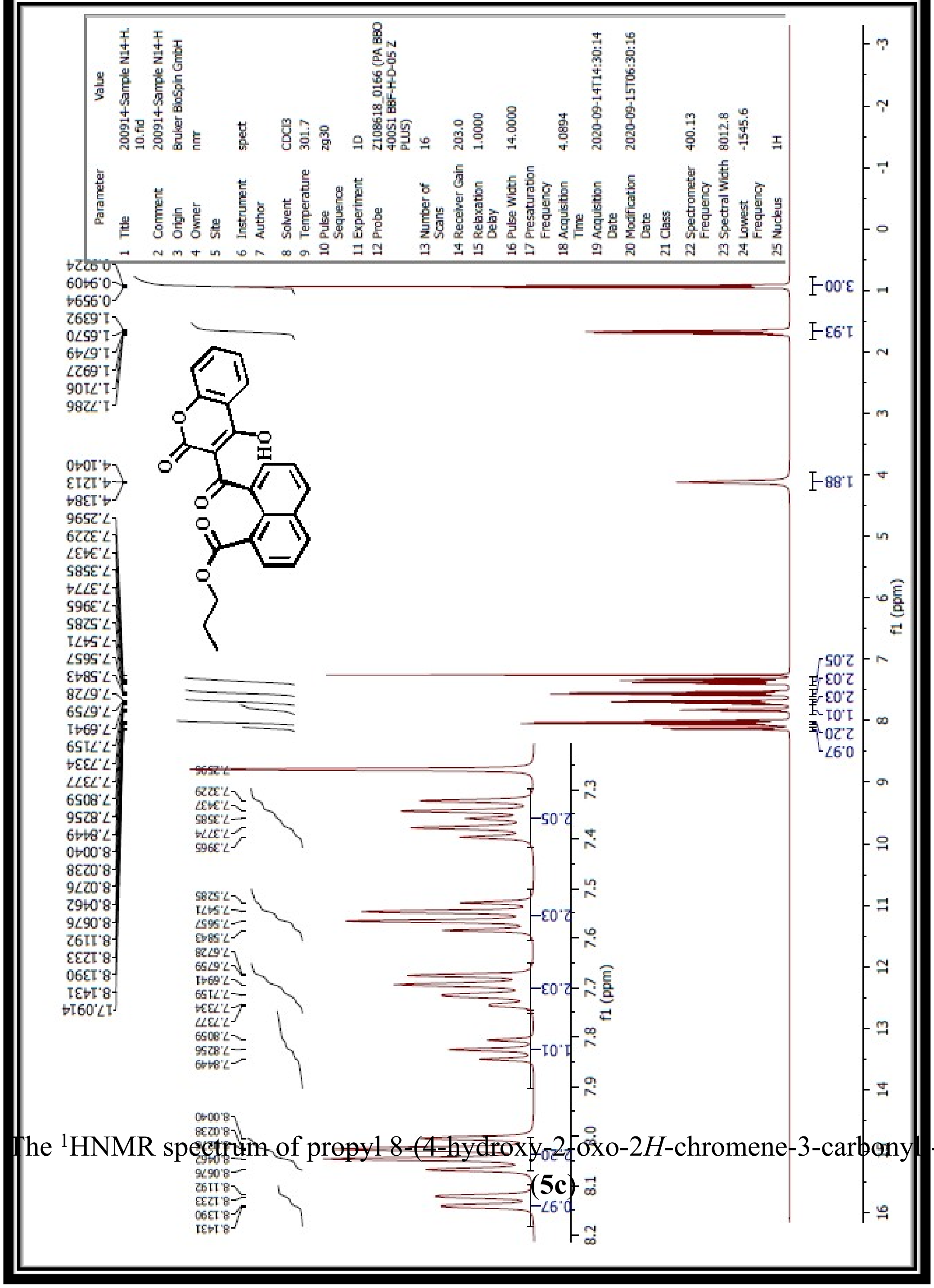

The ^{1}HNMR spectrum of propyl 8-(4-hydroxy-2-oxo-2*H*-chromene-3-carbonyl)-1-naphthoate **(5c)**

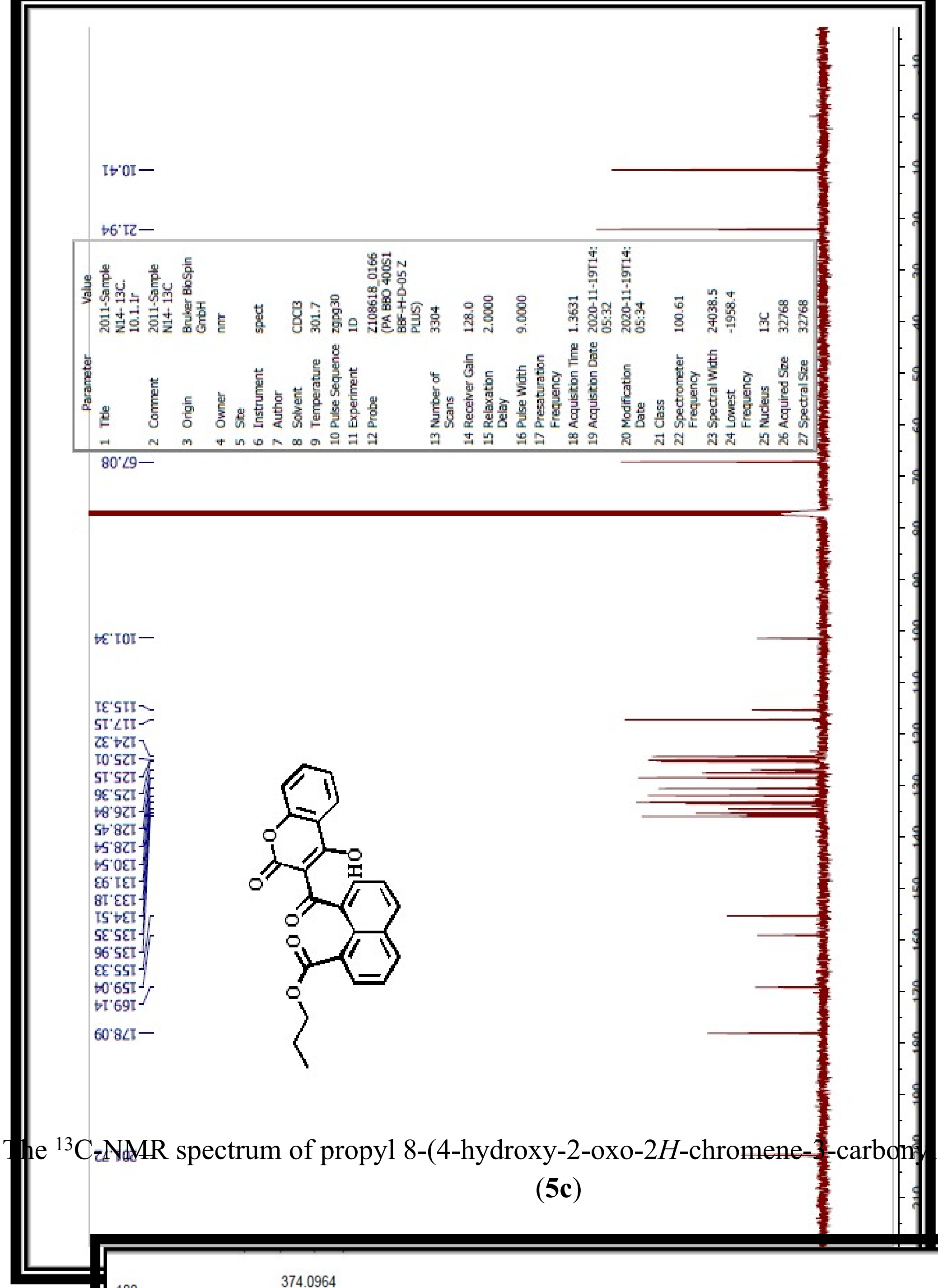

The ^{13}C-NMR spectrum of propyl 8-(4-hydroxy-2-oxo-2*H*-chromene-3-carbonyl)-1-naphthoate (**5c**)

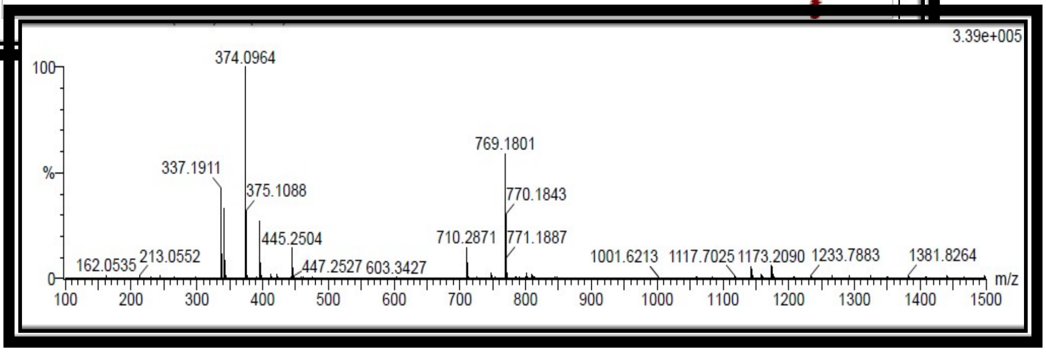

The HRMS spectrum of methyl 8-(2,4-dihydroxyquinoline-3-carbonyl)-1-naphthoate (**5d**)

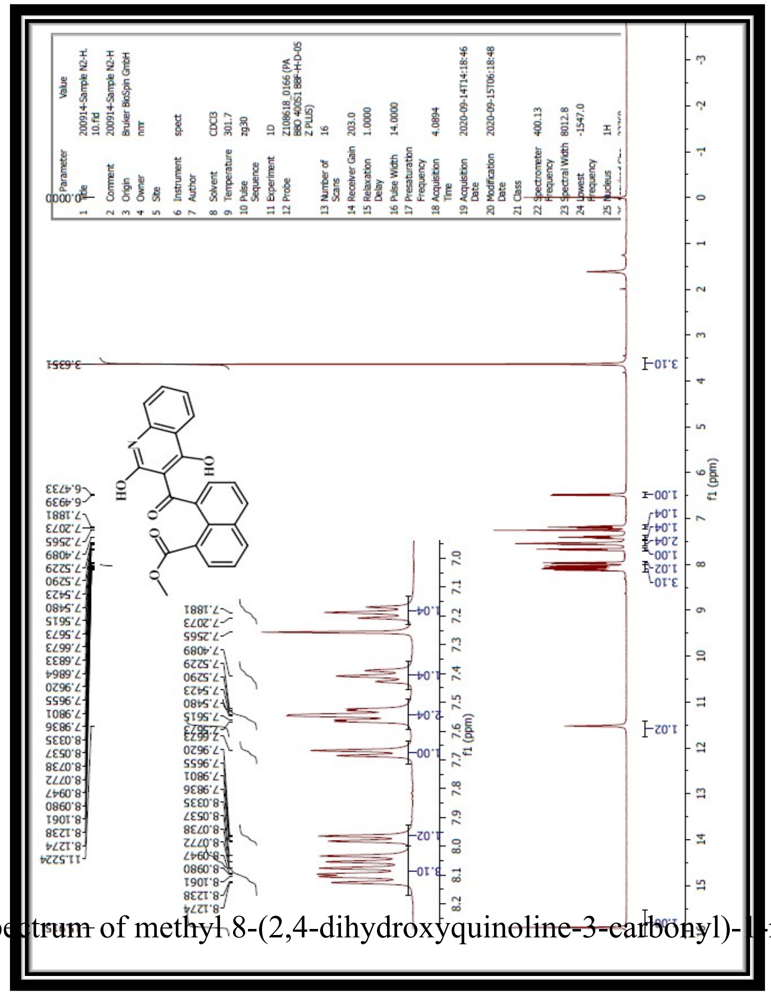

The ^{1}HNMR spectrum of methyl 8-(2,4-dihydroxyquinoline-3-carbonyl)-1-naphthoate (**5d**)

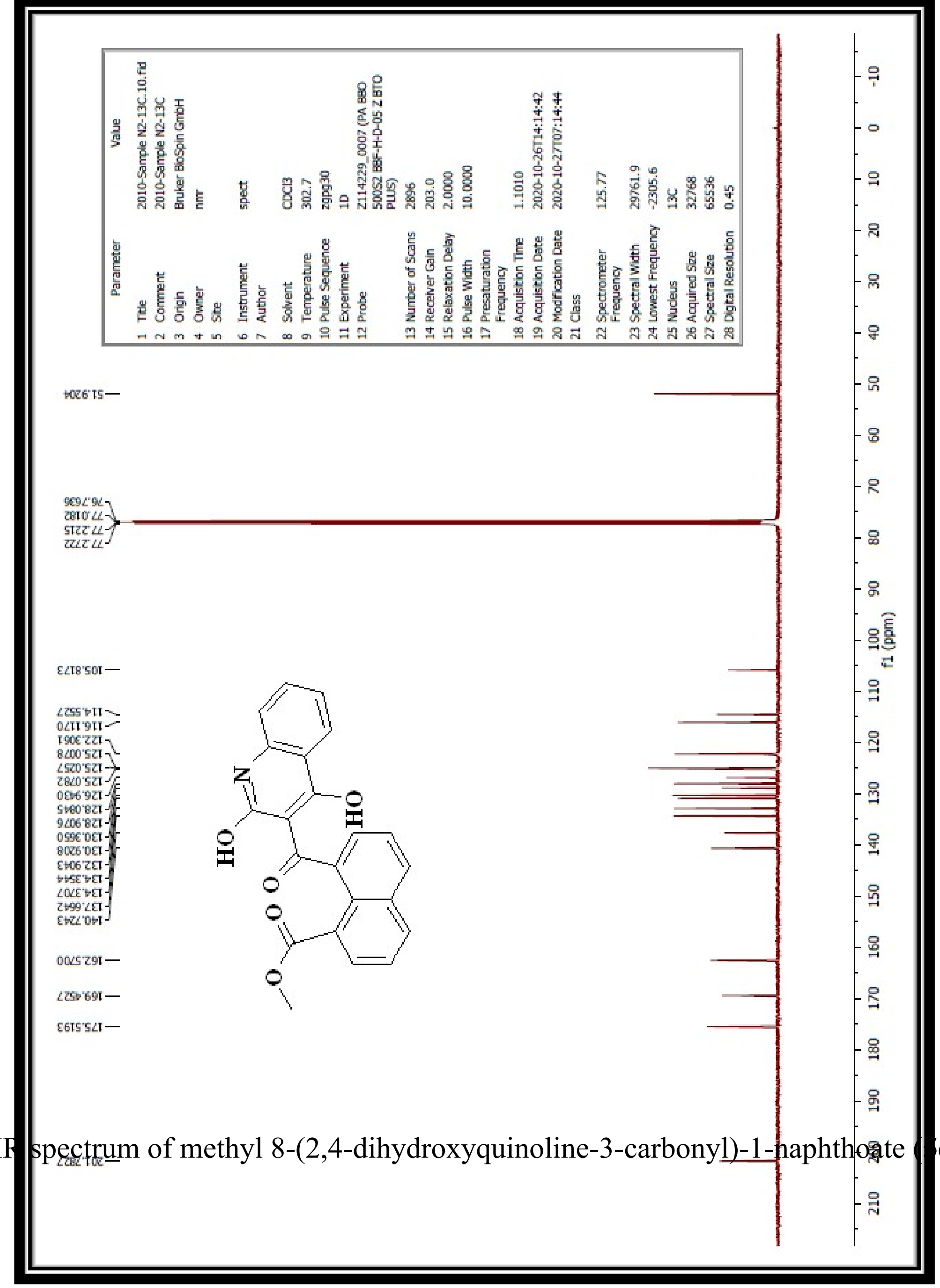

The ^{13}CNMR spectrum of methyl 8-(2,4-dihydroxyquinoline-3-carbonyl)-1-naphthoate ([illegible]**d**)

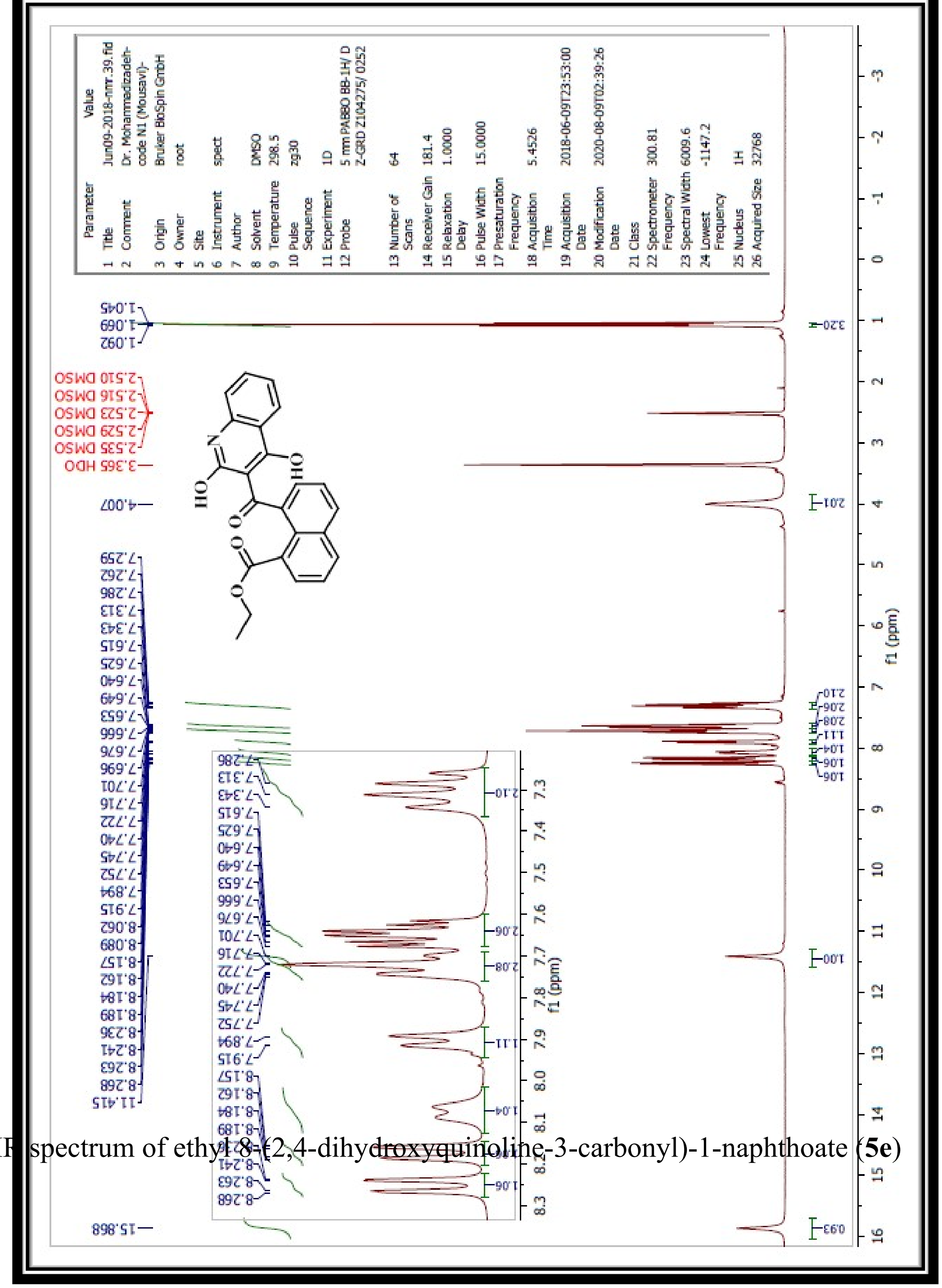

The ^{1}HNMR spectrum of ethyl 8-(2,4-dihydroxyquinoline-3-carbonyl)-1-naphthoate (**5e**)

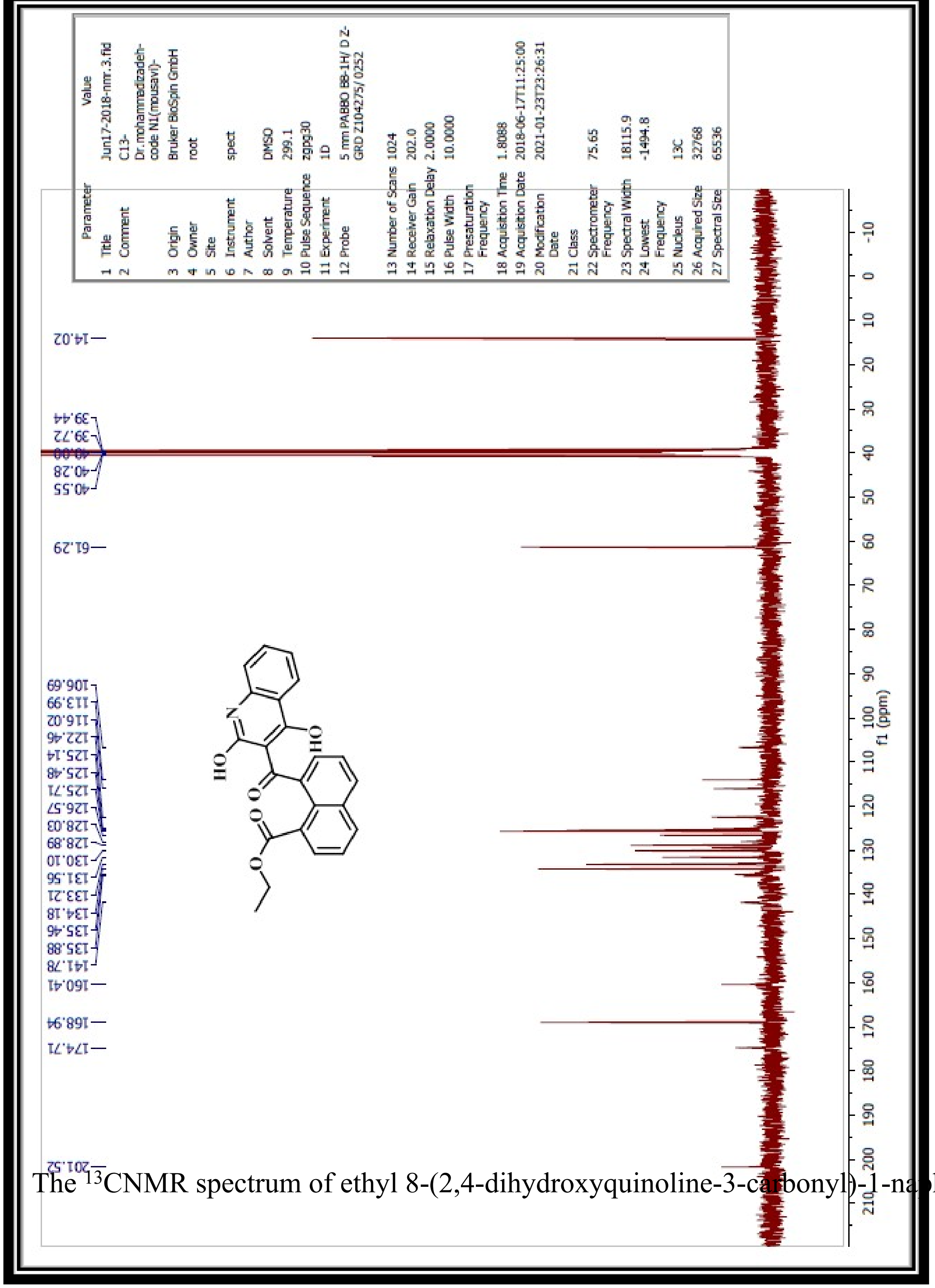

The ^{13}CNMR spectrum of ethyl 8-(2,4-dihydroxyquinoline-3-carbonyl)-1-naphthoate (**5e**)

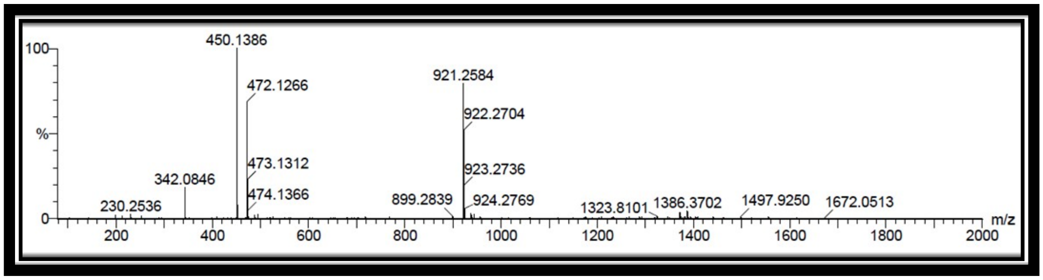

The HRMS spectrum of benzyl 8-(2,4-dihydroxyquinoline-3-carbonyl)-1-naphthoate (**5f**)

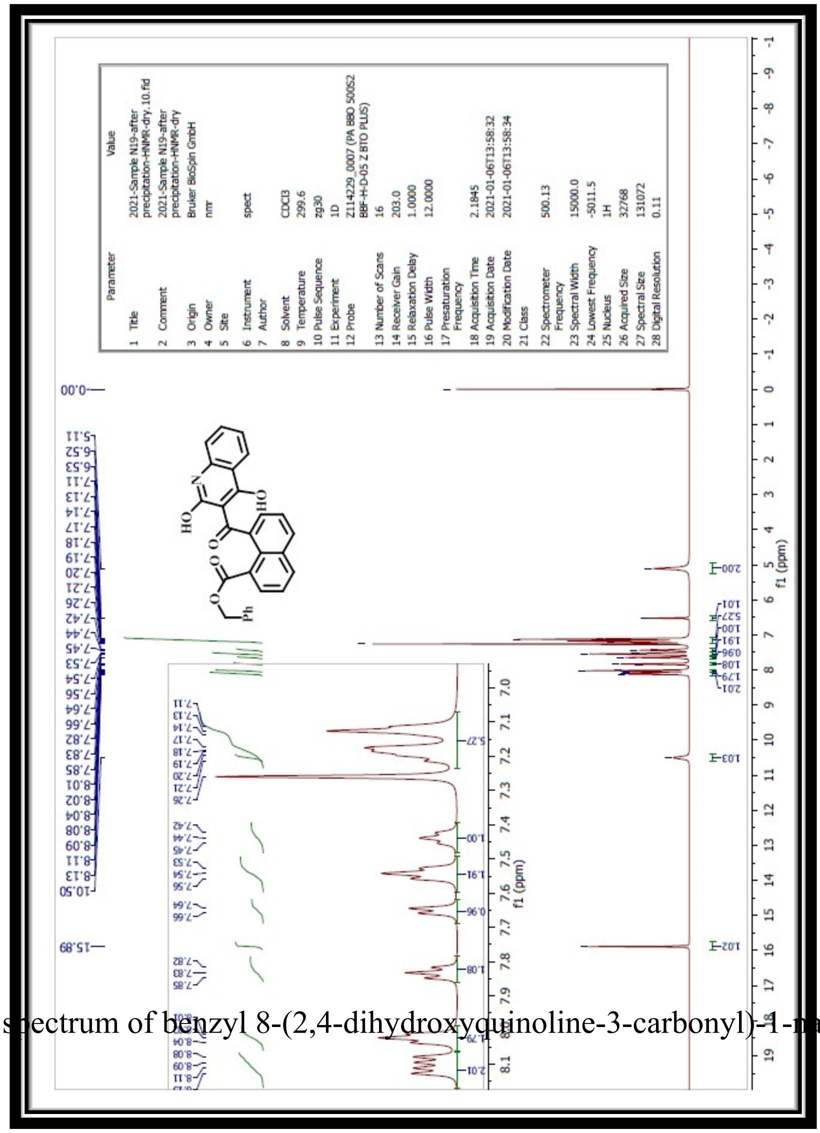

The ^{1}HNMR spectrum of benzyl 8-(2,4-dihydroxyquinoline-3-carbonyl)-1-naphthoate (**5f**)

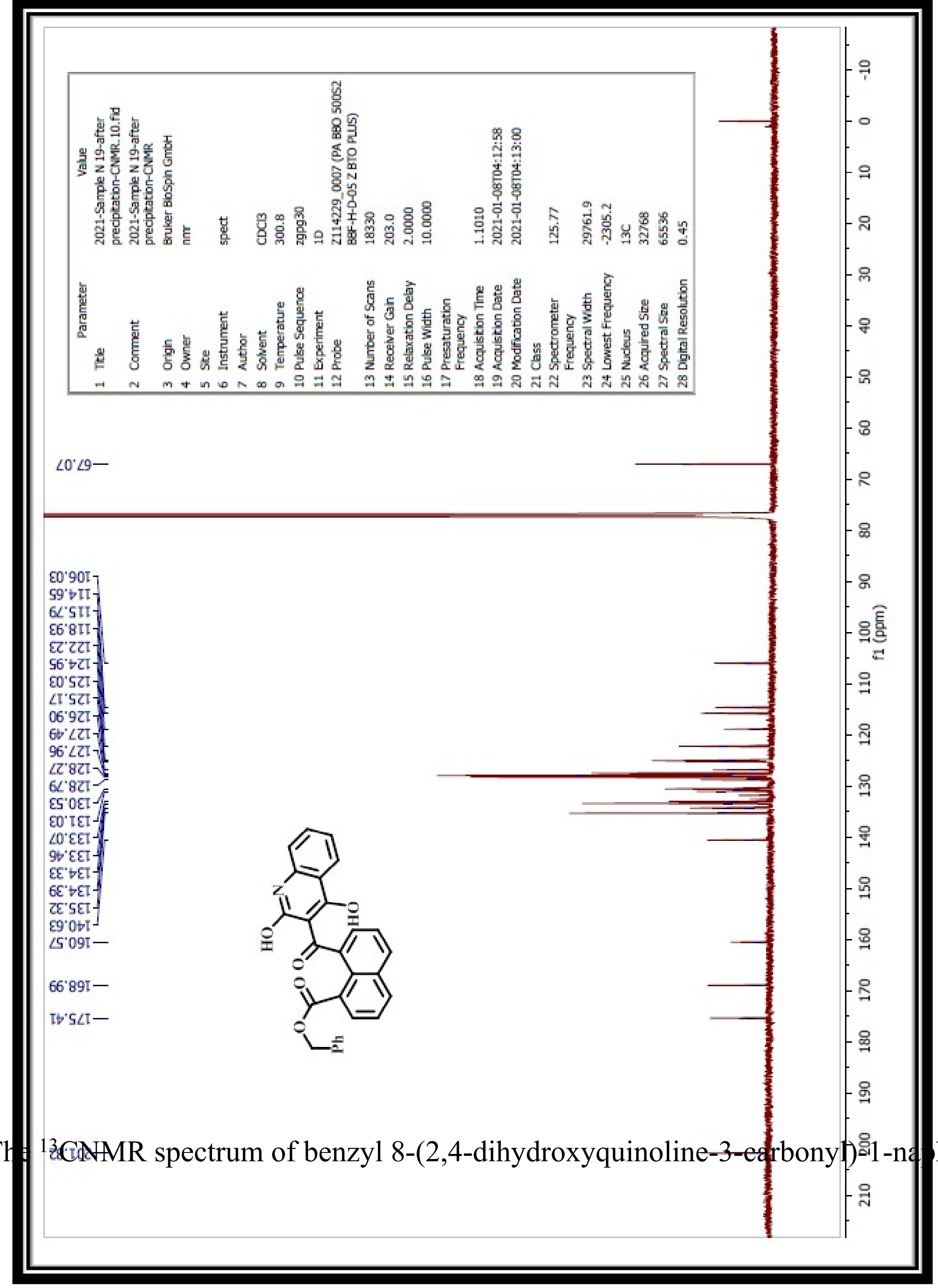

The ^{13}CNMR spectrum of benzyl 8-(2,4-dihydroxyquinoline-3-carbonyl)-1-naphthoate (**5f**)

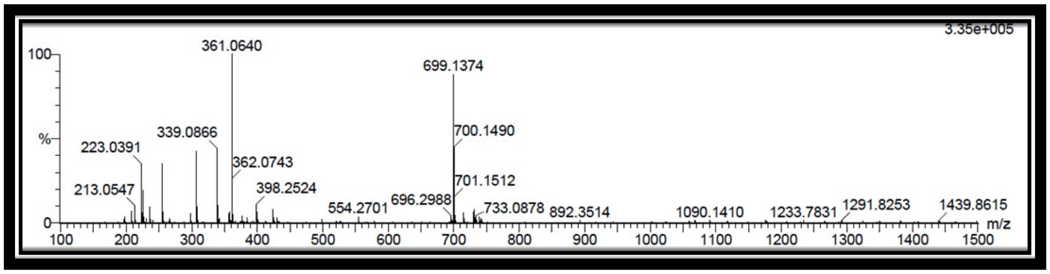

The HRMS spectrum of methyl 8-(4-hydroxy-6-methyl-2-oxo-2H-pyran-3-carbonyl)-1-naphthoate (**5g**)

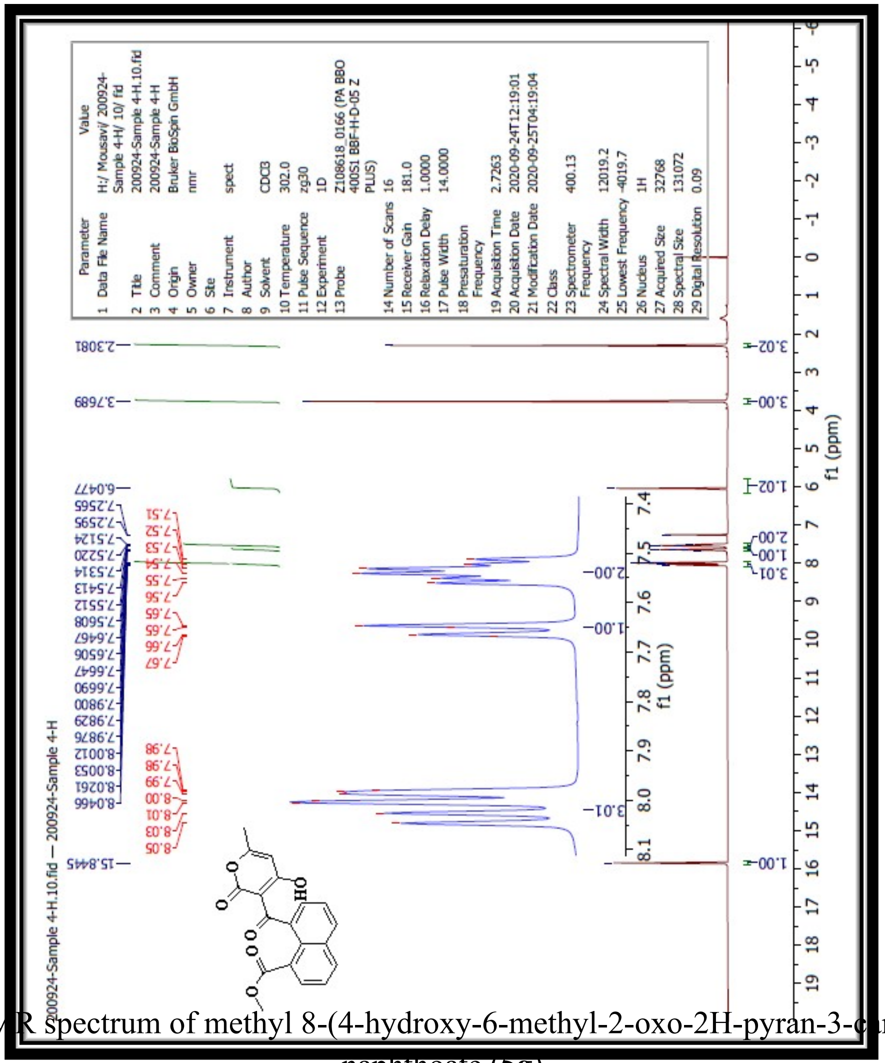

The ^{1}HNMR spectrum of methyl 8-(4-hydroxy-6-methyl-2-oxo-2H-pyran-3-carbonyl)-1-naphthoate (**5g**)

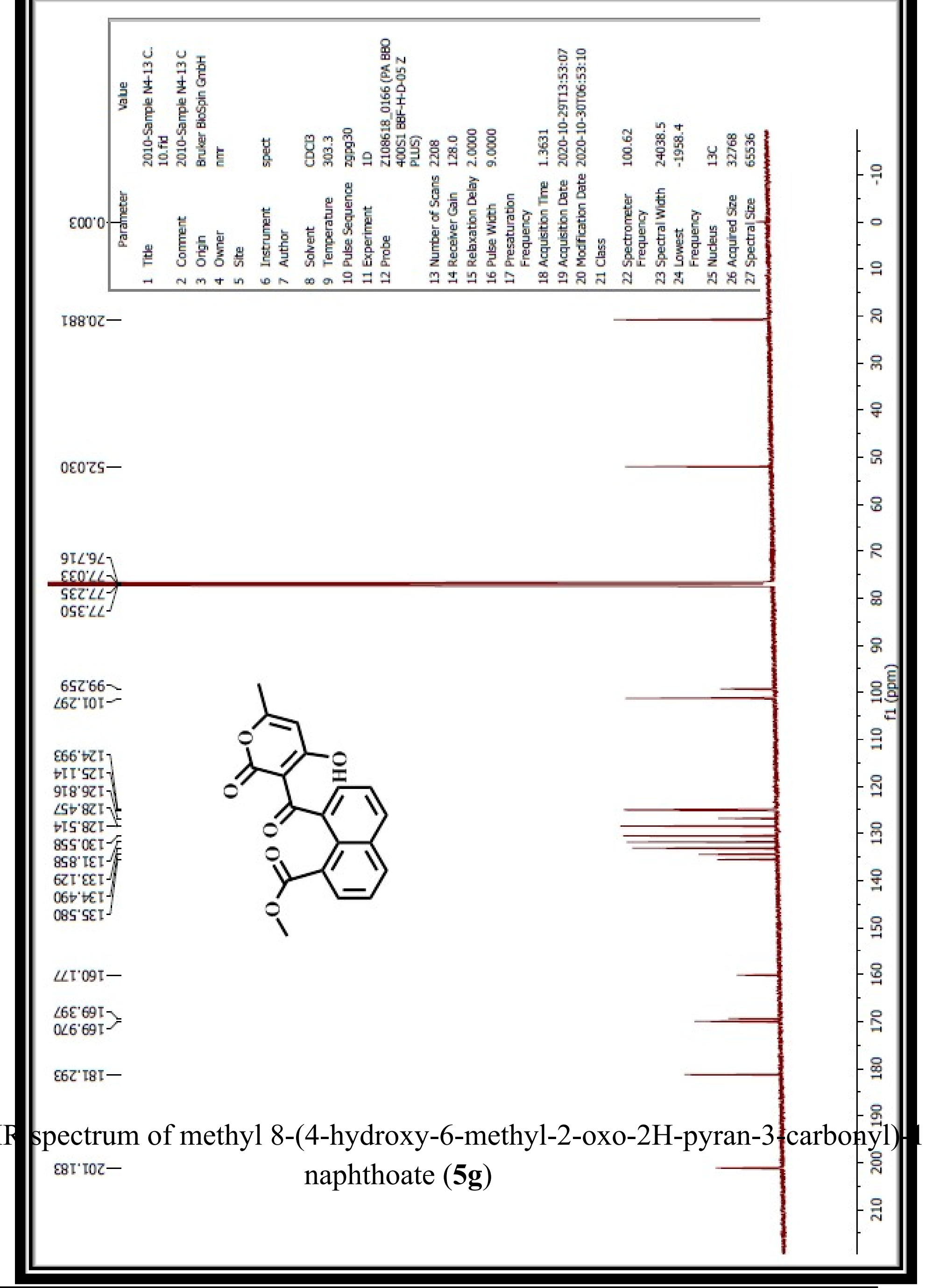

The ^{13}CNMR spectrum of methyl 8-(4-hydroxy-6-methyl-2-oxo-2H-pyran-3-carbonyl)-1-naphthoate (**5g**)

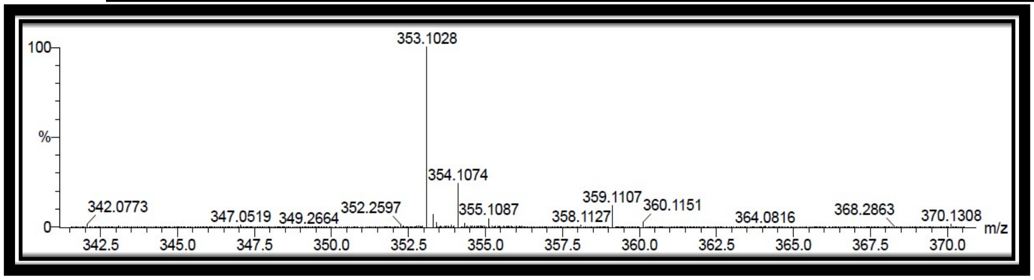

The HRMS spectrum of ethyl 8-(4-hydroxy-6-methyl-2-oxo-2*H*-pyran-3-carbonyl)-1-naphthoate (**5h**)

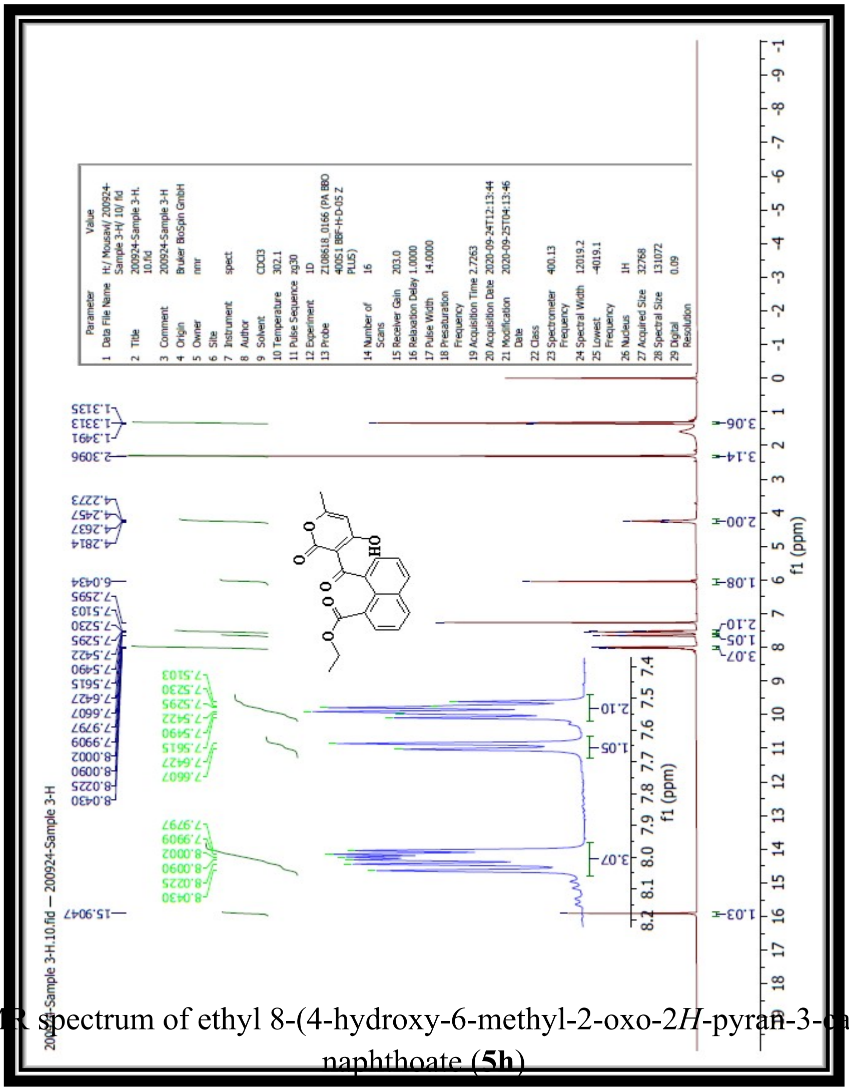

The ^{1}HNMR spectrum of ethyl 8-(4-hydroxy-6-methyl-2-oxo-2*H*-pyran-3-carbonyl)-1-naphthoate (**5h**)

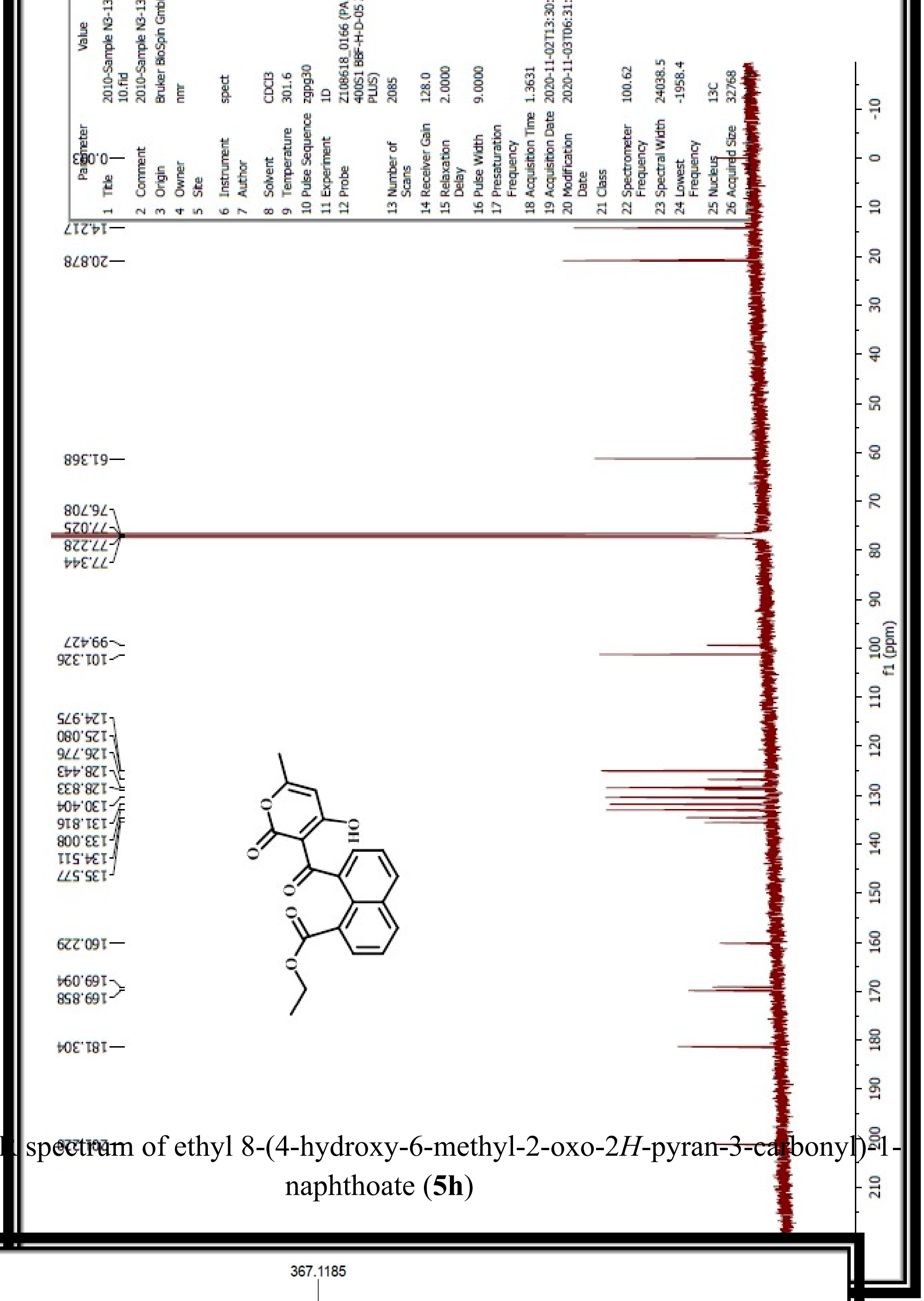

The ^{13}CNMR spectrum of ethyl 8-(4-hydroxy-6-methyl-2-oxo-2*H*-pyran-3-carbonyl)-1-naphthoate (**5h**)

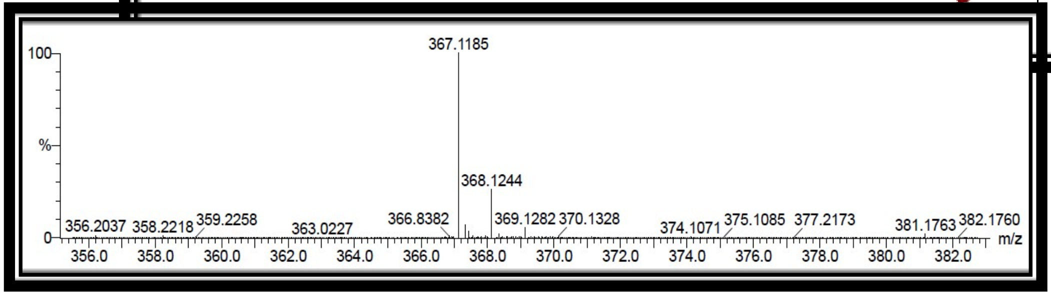

The HRMS spectrum of propyl-8-(4-hydroxy-6-methyl-2-oxo-2*H*-pyran-3-carbonyl)-1-naphthoate (**5i**)

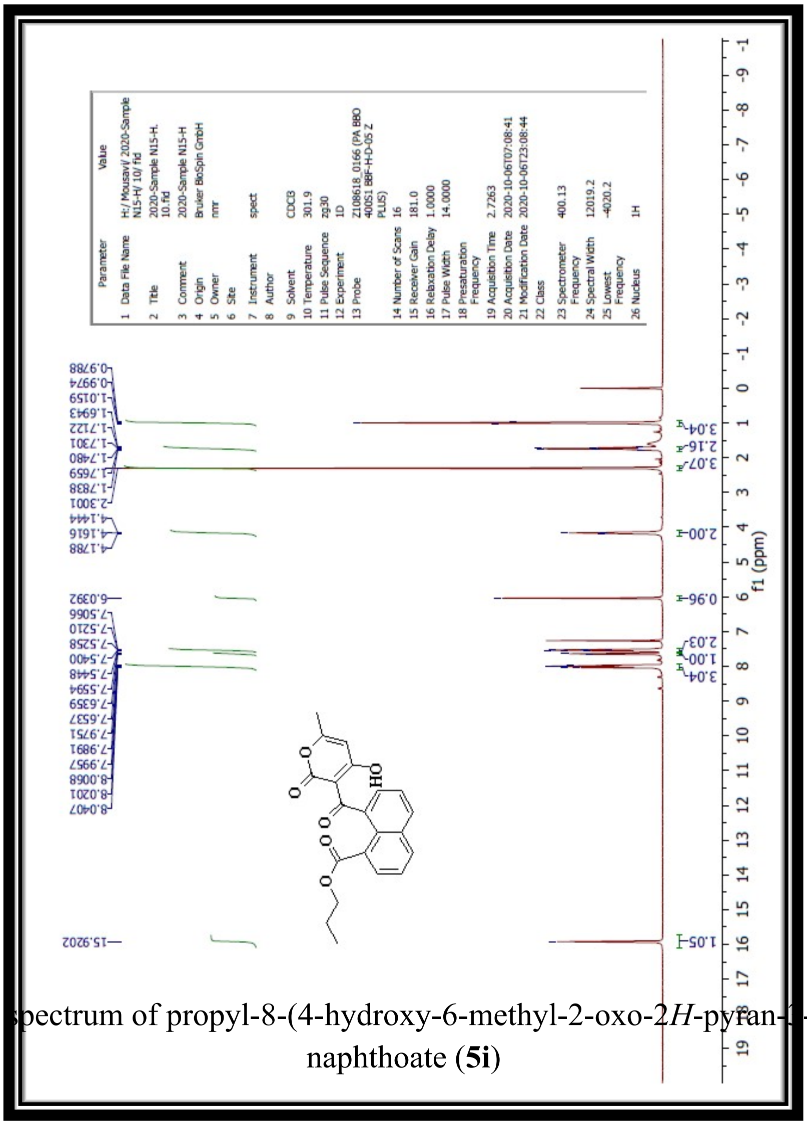

The ^{1}HNMR spectrum of propyl-8-(4-hydroxy-6-methyl-2-oxo-2*H*-pyran-3-carbonyl)-1-naphthoate (**5i**)

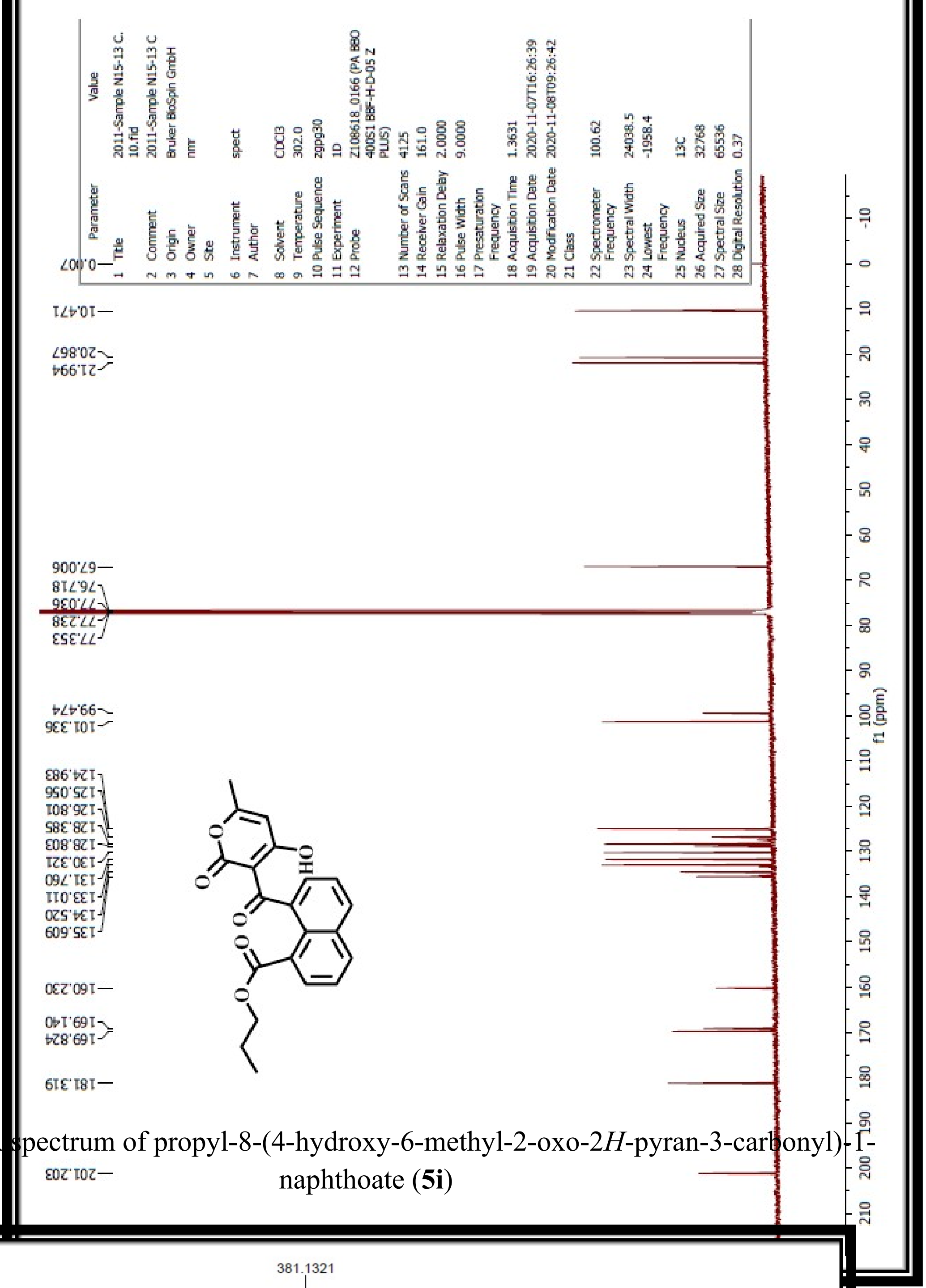

The ^{1}HNMR spectrum of propyl-8-(4-hydroxy-6-methyl-2-oxo-2*H*-pyran-3-carbonyl)-1-naphthoate (**5i**)

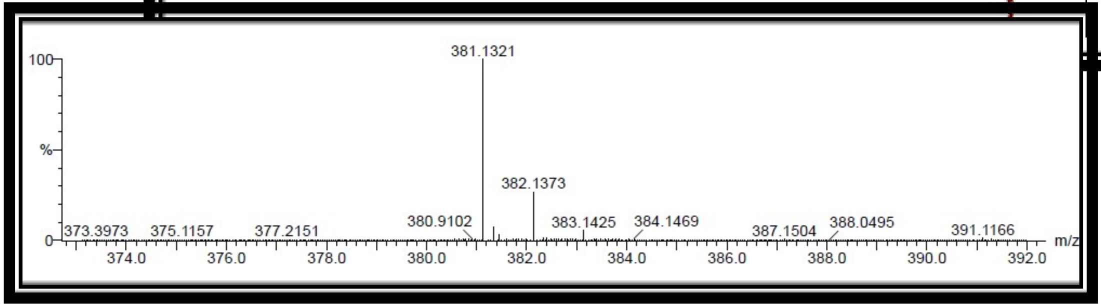

The HRMS spectrum of butyl 8-(4-hydroxy-6-methyl-2-oxo-2*H*-pyran-3-carbonyl)-1-naphthoate (**5j**)

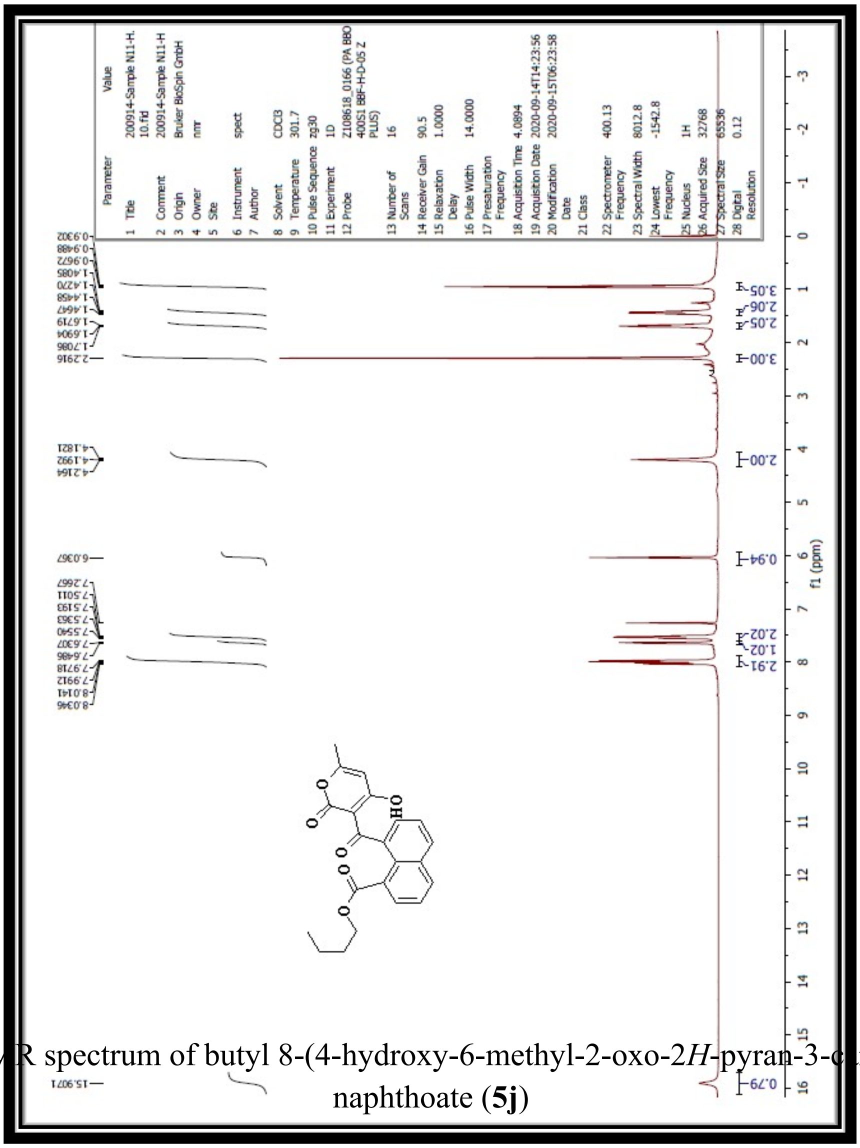

The ^{1}HNMR spectrum of butyl 8-(4-hydroxy-6-methyl-2-oxo-2*H*-pyran-3-carbonyl)-1-naphthoate (**5j**)

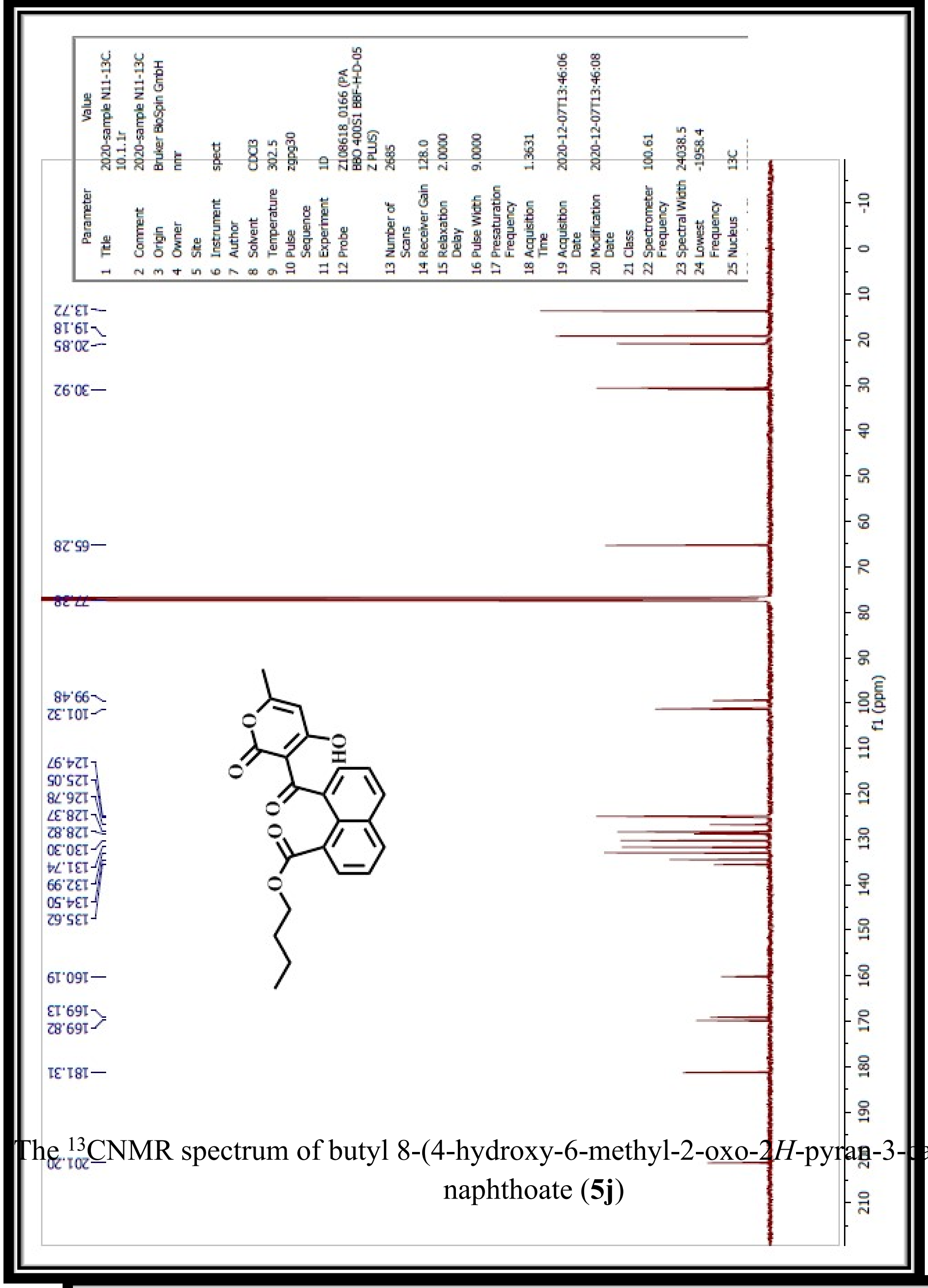

The ^{13}CNMR spectrum of butyl 8-(4-hydroxy-6-methyl-2-oxo-2*H*-pyran-3-carbonyl)-1-naphthoate (**5j**)

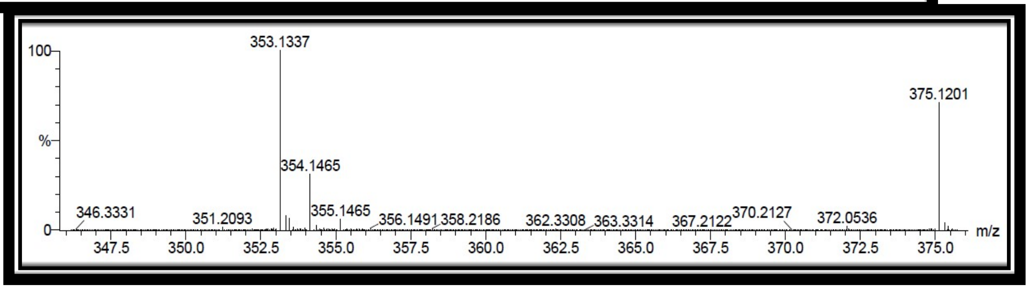

The HRMS spectrum of methyl 8-(2-hydroxy-4,4-dimethyl-6-oxocyclohex-1-ene-1-carbonyl)-1-naphthoate (**5k**)

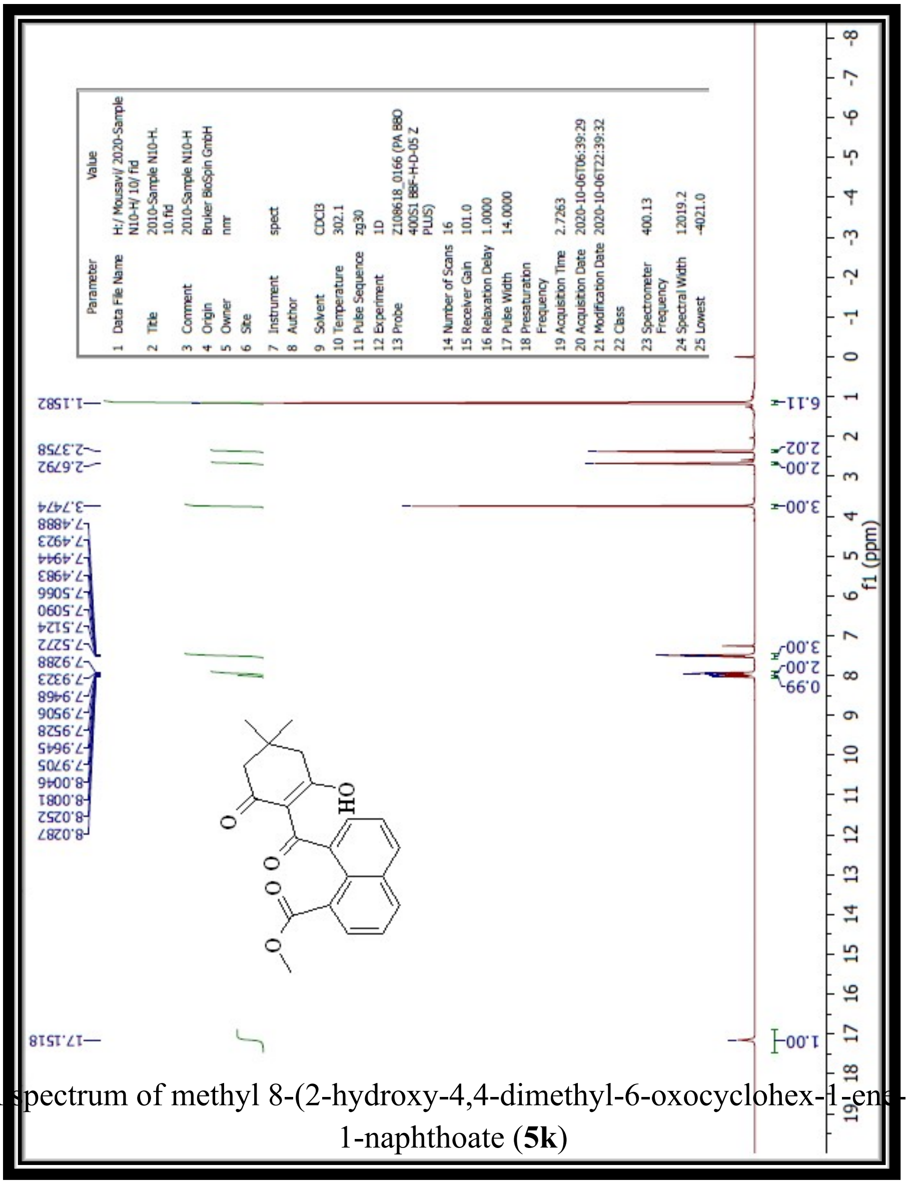

The ^{1}HNMR spectrum of methyl 8-(2-hydroxy-4,4-dimethyl-6-oxocyclohex-1-ene-1-carbonyl)-1-naphthoate (**5k**)

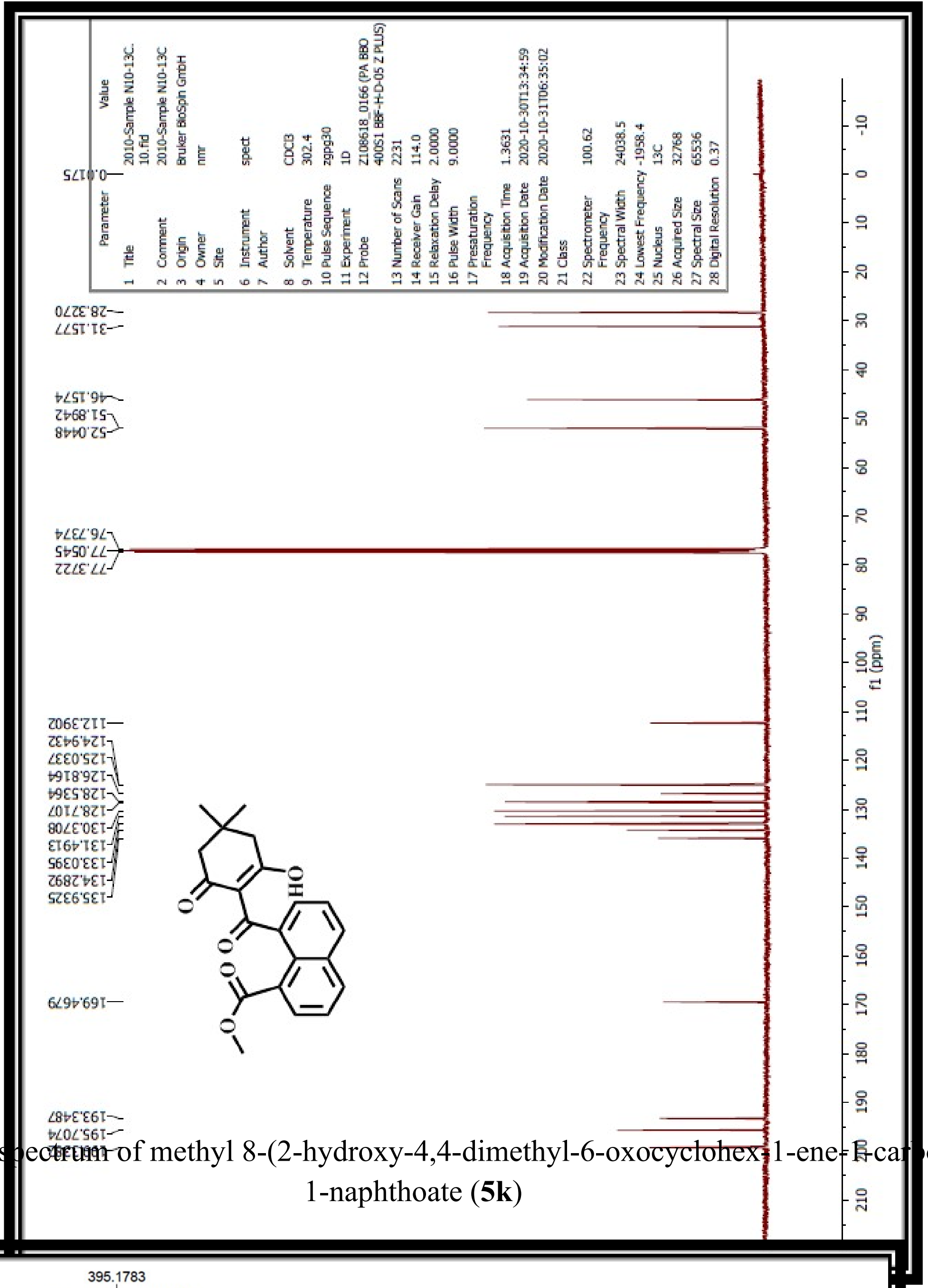

The ^{13}CNMR spectrum of methyl 8-(2-hydroxy-4,4-dimethyl-6-oxocyclohex-1-ene-1-carbonyl)-1-naphthoate (**5k**)

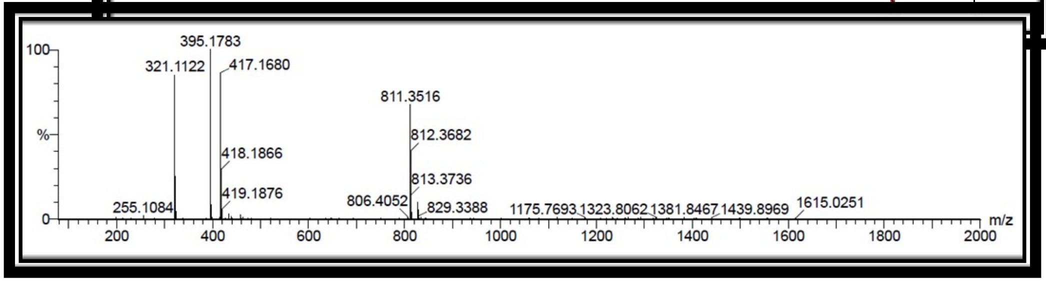

The HRMS spectrum of butyl 8-(2-hydroxy-4,4-dimethyl-6-oxocyclohex-1-ene-1-carbonyl)-1-naphthoate (**5l**)

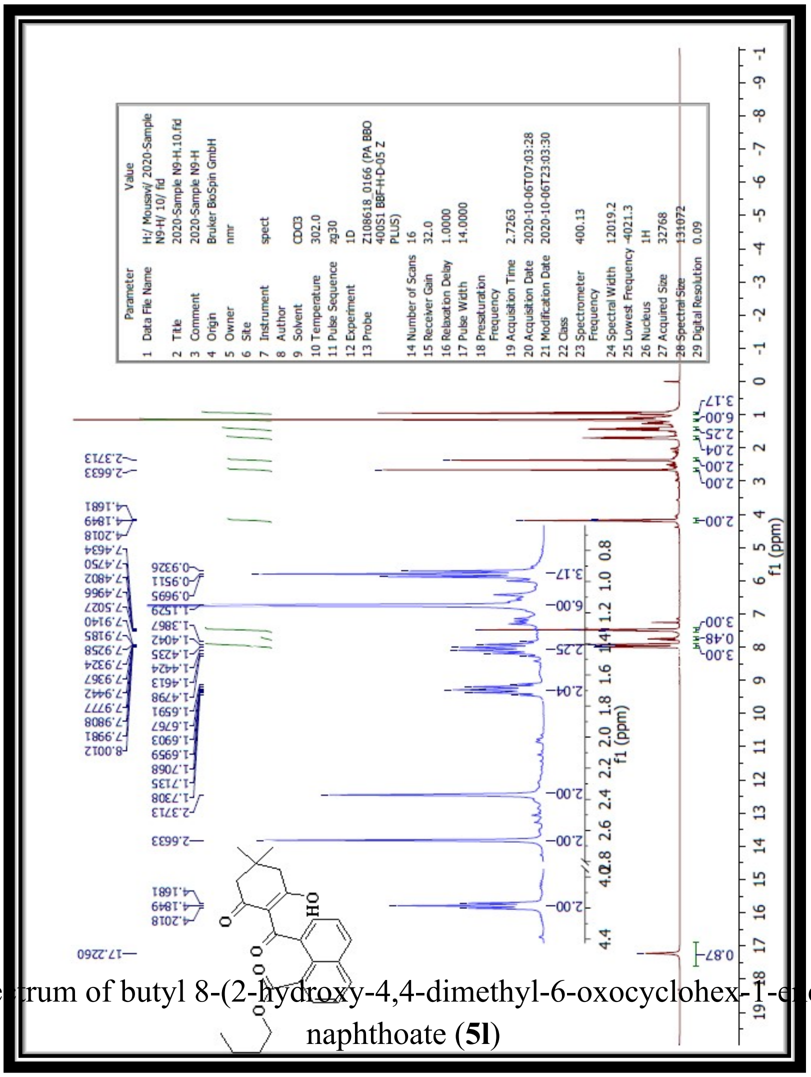

The ^{1}HNMR spectrum of butyl 8-(2-hydroxy-4,4-dimethyl-6-oxocyclohex-1-ene-1-carbonyl)-1-naphthoate (**5l**)

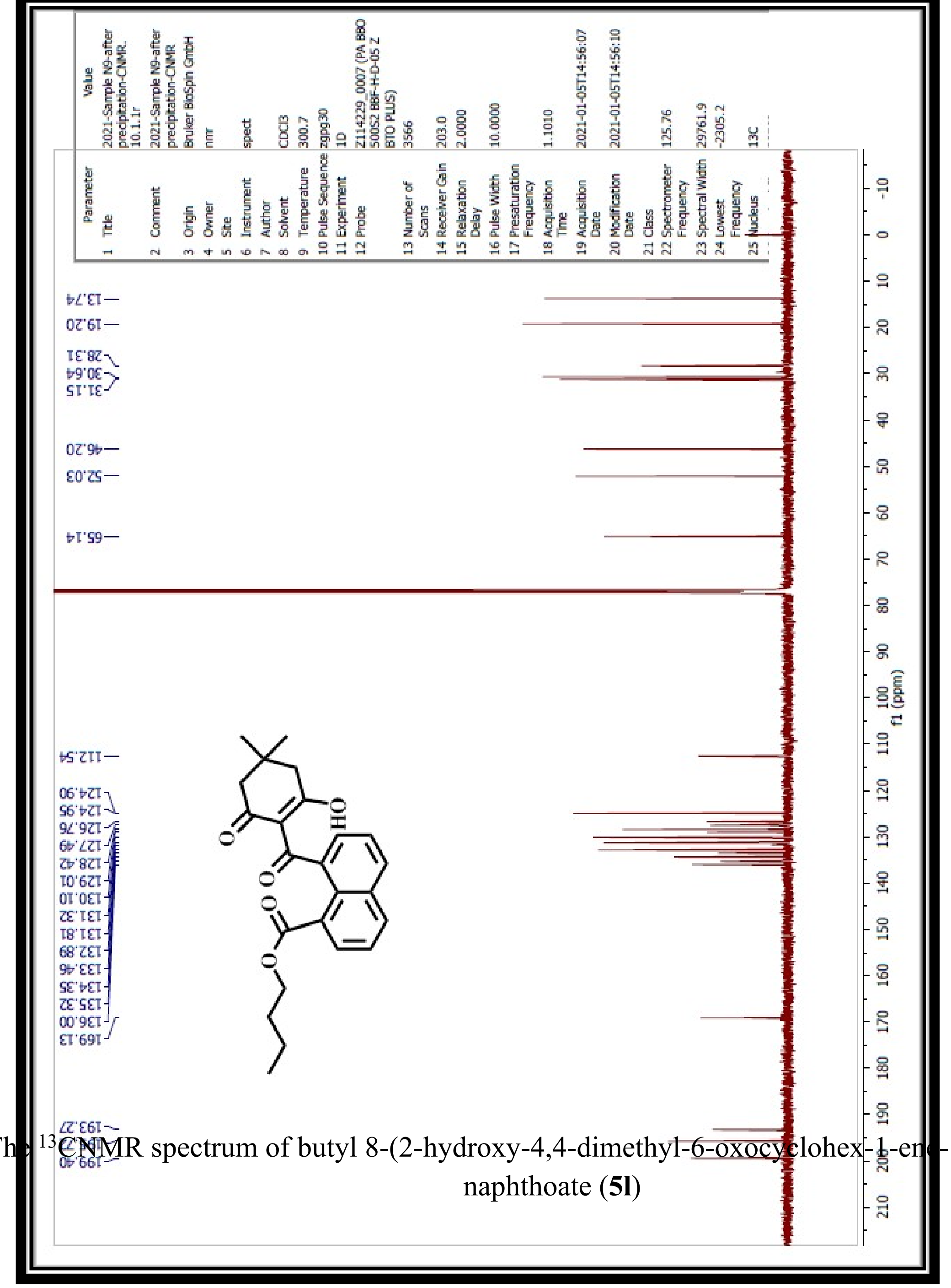

The ^{13}C NMR spectrum of butyl 8-(2-hydroxy-4,4-dimethyl-6-oxocyclohex-1-ene-1-carbonyl)-1-naphthoate (**5l**)
